# Bidirectional hybrid erythritol-inducible promoter for synthetic biology in *Yarrowia lipolytica*

**DOI:** 10.1186/s12934-023-02020-6

**Published:** 2023-01-12

**Authors:** Lea Vidal, Esteban Lebrun, Young-Kyoung Park, Guillaume Mottet, Jean-Marc Nicaud

**Affiliations:** 1 UMR1319, Team BIMLip: Integrative Biology of Microbial Lipid Metabolism, Université Paris-Saclay, INRAE, AgroParisTech, Micalis Institute, Domaine de Vilvert, 78350 Jouy-en-Josas, France; 2 Large Molecules Research, Sanofi, 94400 Vitry-Sur-Seine, France

**Keywords:** Bidirectional promoter, Inducible, Erythritol, Hybrid promoter, Co-expression, *Yarrowia lipolytica*, Synthetic biology

## Abstract

**Background:**

The oleaginous yeast *Yarrowia lipolytica* is increasingly used as a chassis strain for generating bioproducts. Several hybrid promoters with different strengths have been developed by combining multiple copies of an upstream activating sequence (UAS) associated with a TATA box and a core promoter. These promoters display either constitutive, phase-dependent, or inducible strong expression. However, there remains a lack of bidirectional inducible promoters for co-expressing genes in *Y. lipolytica*.

**Results:**

This study built on our previous work isolating and characterizing the UAS of the erythritol-induced genes *EYK1* and *EYD1* (UAS-eyk1). We found an erythritol-inducible bidirectional promoter (BDP) located in the *EYK1*-*EYL1* intergenic region. We used the BDP to co-produce YFP and RedStarII fluorescent proteins and demonstrated that the promoter’s strength was 2.7 to 3.5-fold stronger in the *EYL1* orientation compared to the *EYK1* orientation. We developed a hybrid erythritol-inducible bidirectional promoter (HBDP) containing five copies of UAS-eyk1 in both orientations. It led to expression levels 8.6 to 19.2-fold higher than the native bidirectional promoter. While the BDP had a twofold-lower expression level than the strong constitutive TEF promoter, the HBDP had a 5.0-fold higher expression level when oriented toward *EYL1* and a 2.4-fold higher expression level when oriented toward *EYK1*. We identified the optimal media for BDP usage by exploring yeast growth under microbioreactor conditions. Additionally, we constructed novel Golden Gate biobricks and a destination vector for general use.

**Conclusions:**

In this research, we developed novel bidirectional and hybrid bidirectional promoters of which expression can be fine-tuned, responding to the need for versatile promoters in the yeast *Y. lipolytica*. This study provides effective tools that can be employed to smoothly adjust the erythritol-inducible co-expression of two target genes in biotechnology applications. BDPs developed in this study have potential applications in the fields of heterologous protein production, metabolic engineering, and synthetic biology.

**Supplementary Information:**

The online version contains supplementary material available at 10.1186/s12934-023-02020-6.

## Background

Over recent years, the oleaginous yeast species *Yarrowia lipolytica* has become an efficient chassis for producing a wide variety of biomolecules, including heterologous proteins [1–3], organic acids [4, 5], erythritol [6, 7], and aroma compounds [8–10], as well as unusual lipids and lipid derivatives, including from organic wastes [11] (for reviews, see [12] and [13]).

Increasing interest in *Y. lipolytica* has led to numerous dedicated tools for enhancing its production potential. These tools include Golden Gate assembly (GGA), which allows one-step cloning of up to three transcription units into various integrative vectors [14]. GGA has a library of genetic parts, which is constantly being enriched. Furthermore, CRISPR-Cas9 technology has been adapted for the genetic engineering of *Y. lipolytica* [15–17], and a single-cell screening method based on droplet microfluidics has been developed [18]. Lastly, several promoter sequences have been characterized and engineered to fine-tune gene expression in *Y. lipolytica*, providing a bank of natural and synthetic promoters that display constitutive, phase-dependent, or inducible expression [19–22]. While a variety of synthetic biology tools have been applied in *Y. lipolytica*, see [12, 23–25], genetic parts—notably promoters—are still lacking if the goal is robust gene expression at industrial scales.

**Fig. 1:**
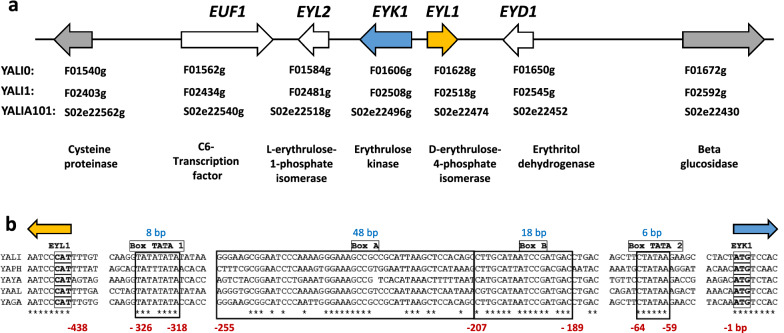
Genomic structure of the erythritol utilization cluster and multiple alignment of pEYK450. **a** Erythritol catabolism gene cluster in *Yarrowia lipolytica.* Genes involved in erythritol catabolism are clustered on chromosome F. *EUF1* (YALI0F01562g) encodes a transcription factor; *EYL2* (YALI0F01584g) encodes L-erythrulose-1-phosphate isomerase; *EYK1* (YALI0F01606g) encodes erythrulose kinase; *EYL1* (YALI0F01628g) encodes D-erythrulose-4-phosphate; and *EYD1* (YALI0F01650g) encodes the erythritol dehydrogenase. The border genes YALI0F01540g and YALI0F01672g code for a cysteine proteinase and a beta-glucosidase, respectively. Arrow color indicates the following: white = gene involved in erythritol utilization; gray = flanking genes; blue = EYK1; and orange = EYL1. The conventional genes’ names are shown for three *Y. lipolytica* strains — E150 (YALI0), W29 (YALI1), and A101 (YALIA101). **b** Partial alignment of conserved motifs in the putative bidirectional promoter pEYK450, located between the divergent *EYL1* and *EYK1* genes. Outlined in black are the conserved blocks corresponding to elements involved in *EYK1* expression, including induction by erythritol and erythrulose. The genomic sequences are from *Yarrowia lipolytica* W29 (YALI), *Y. phangngensis* (YAPH), *Y. yakushimensis* (YAYA), *Y. alimentaria* (YAAL), and *Y. galli* (YAGA). The in-boxes CAT and ATG are the start codons of *EYL1* and *EYK1*, respectively. Boxes TATA 1 and TATA 2 correspond to the putative TATA boxes for *EYL1* and *EYK1,* respectively. Boxes A and B correspond to the sequences of UAS1-eyk1 and UAS2-eyk1, respectively. *EYK1*´s start codon is the reference for motif locations. (Adapted from Mirończuk et al*.* [29] and Trassaert et al*.* [21])

Recently, the metabolic dynamics of erythritol catabolism in *Y. lipolytica* have been characterized [26, 27]. The first step of the pathway is catalyzed by an erythritol dehydrogenase encoded by *EYD1* (YALI0F01650g), which converts erythritol into erythrulose. Second, erythrulose is phosphorylated by an erythrulose kinase encoded by *EYK1* (YALI0F01606g). Third, L-erythrulose-1P is converted into D-erythrulose-4P by an L-erythrulose-1P isomerase encoded by *EYL2* (YALI0F01584g). The result is then transformed into erythrose-4P by a D-erythrulose-4P isomerase encoded by *EYL1* (YALI0F01628g). It has been suggested that *EUF1* (YALI0F01562g) would code for a transcription factor involved in erythritol utilization [28]. All five genes mentioned above are located in *Y. lipolytica*’s chromosome F in a region called the erythritol utilization cluster [29, 30] (Fig. 1a), and their expression can be induced by erythritol as well as by erythrulose.

Recently, putative upstream activation sequences (UASs) associated with *EYK1* and *EYD1* have been characterized. Such has been accomplished by identifying their conserved regulatory motifs (CRM) via phylogenetic footprinting and site-directed mutagenesis. Furthermore, yellow fluorescent protein (YFP) and RedStarII fluorescent protein have been used as reporters to reveal expression patterns of native and erythritol-induced promoters [21, 22]. The structure of *EYK1*’s promoter (pEYK) has been elucidated (Fig. 1b), as well as its inducibility by erythritol and erythrulose, which is led by UAS1-eyk1 (also called Box A). Additionally, we have previously developed several erythritol-inducible hybrid promoters that include UAS1-eyk1, allowing the fine-tuning of heterologous gene expression in *Y. lipolytica* [21, 22].

Further analyses of the erythritol catabolism gene cluster suggested that pEYK could be not only *EYK1*’s promoter but also *EYL1*’s promoter through a mechanism of divergent transcription, making pEYK a bidirectional promoter (BDP).

A BDP is a DNA sequence that allows DNA transcription in both directions, a process that is also known as divergent transcription. BDPs are common in nature and can be composed of either one core promoter or two outward-facing core promoters (for a review, see [31]). BDPs allow the accurate co-regulation of expression for two genes via a mechanism that does not use internal ribosome entry sites [32, 33]. Moreover, BDP usage increases the evolutionary stability of synthetic genetic circuits [34], a trait of great interest for strains with industrial applications like *Y. lipolytica*. Recently, three of *Y. lipolytica*’s BDPs were identified and engineered [35]. However, because these BDPs are regulated by copper, they cannot be employed in industrial settings, given copper’s toxicity and the resulting bioremediation issues.

In this study, we engineered a new erythritol-inducible hybrid BDP for *Y. lipolytica*. We constructed natural and hybrid BDP biobricks using pEYK and made them GGA-compatible. Expression was characterized in both transcription orientations using reporter fluorescent proteins (YFP and RedStarII), and we assessed the strongest hybrid promoter’s behavior under high-density cultivation conditions.

## Results

### Erythritol catabolism cluster contains a putative bidirectional promoter

Given the pronounced interest in BDPs for gene co-transcription and synthetic biology applications, we explored the dynamics of an inducible BDP in the erythritol catabolism gene cluster in *Y. lipolytica*. Three pairs of divergent genes could be identified in the cluster: YALI0F01540g and *EUF1*, *EYK1* and *EYL1*, and *EYD1* and YALI0F01672g. To determine which intergenic regions could include a BDP, we annotated and compared the cluster sequences from three *Y. lipolytica* strains. E150 is the first sequenced *Y. lipolytica* strain [36]; W29 is the French wild-type strain [37] from which every strain in our laboratory has been derived; A101 is the Polish wild-type strain used by W. Rymowicz’s research group for developing erythritol-producing strains [38]. The analysis of the sequences revealed several differences in amino acids (Additional file 1: annotated sequence of the erythritol locus in the E150 strain; Additional file 2: annotated sequence of the erythritol locus in the W29 strain; Additional file 3: annotated sequence of the erythritol locus in the A101 strain). Furthermore, sequence alignment revealed that YALIA101S02e22430 (in A101 strain) should have the same start codon as YALI0F01672g (in E150 strain) and YALI1F02592g (in W29 strain), highlighting that the start codons of YALI0F01672g and YALI1F02592g may have been incorrectly predicted and annotated in the GRYC database.

More importantly, the analysis showed that the region between *EYK1* and *EYL1* is 438 bp long, while the regions between YALI0F01540g and *EUF1* and between *EYD1* and YALI0F01672g are 3,178 bp and 4,649 bp long, respectively (Fig. 1a). In eukaryotes, UASs are located around − 1000/ + 50 bp from the transcription start site [39]. Along with the evolutionary pressures acting on BDPs [40], we predicted that the region between *EYK1* and *EYL1* would be the only one in the cluster to potentially contain a BDP.

The intergenic region between *EYK1* and *EYL1* was studied in past research focusing solely on pEYK [21, 22]. It was shown that pEYK is erythritol inducible, and one TATA box was identified near the *EYL1* start codon. To evaluate the hypothesis of pEYK being a BDP, we aligned the DNA sequences of the intergenic region coming from five species of the genus *Yarrowia* (*Y. lipolytica* W29, *Y. phangngensis*, *Y. yakushimensis*, *Y. alimentaria*, and *Y. galli*). We identified a second TATA box near the *EYK1* start codon. The two potential TATA boxes were found relatively close to the beginning of *EYK1* (Box TATA 2, − 64–59) and *EYL1* (Box TATA 1, − 326–318) (Fig. 1b).

### Region between *EYK1* and *EYL1* is an erythritol-inducible BDP

**Fig. 2:**
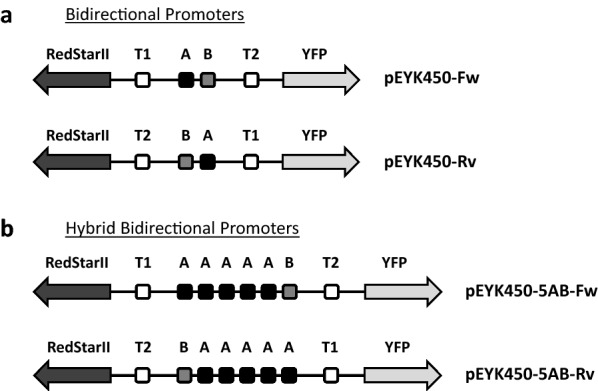
Schematic representation of the bidirectional and hybrid bidirectional promoters used in this study. **a** Wild-type promoter pEYK450 in forward (above) and reverse (below) transcription orientations within a reporter expression cassette. T1, T2, A, and B are Box TATA 1, Box TATA 2, Box A, and Box B, respectively. **b** Hybrid promoter pEYK450-5AB in forward (above) and reverse (below) transcription orientations within a reporter expression cassette. It contains five copies of Box A. Black and gray arrows are *RedStarII* and *YFP* reporter genes, respectively

To assess whether the *EYK1*-*EYL1* intergenic region contained a BDP, we constructed a reporter system relied on two reporter genes coding for yellow and red fluorescent proteins (YFP and RedStarII, respectively). Reporter expression cassettes were constructed with BDPs or hybrid BDPs (HBDPs) in both transcription orientations (Fig. 2).

**Fig. 3:**
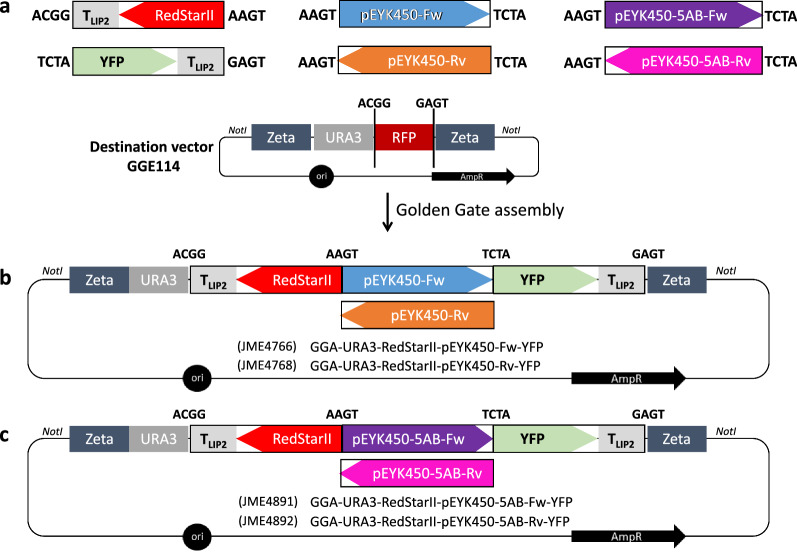
Schematic representation of the Golden Gate assembly strategy used in this study. **a** Destination vector GGE114 and biobricks of the wild-type promoter pEYK450 (forward or reverse transcription), the hybrid promoter pEYK450-5AB containing five copies of Box A (forward or reverse transcription), and the fluorescent reporter genes (*RedStarII* or *YFP* ending with T_LIP2_ terminator). The letters on both sides of the biobricks represent the four-nucleotide overhangs generated by the *Bsa*I restriction sites. The destination vector contains Zeta regions for the specific integration of the expression cassette into the *Y. lipolytica*’s genome, the *URA3* marker for yeast transformants selection, and the chromoprotein RFP surrounded by GGA sticky *Bsa*I sites for *E. coli* transformants selection. The expression cassette is releasable by *Not*I digestion to promote yeast transformation. **b** Assembled plasmids containing both transcription orientations of pEYK450. Plasmid names are indicated. **c** Assembled plasmids containing both transcription orientations of pEYK450-5AB. Plasmid names are indicated. The different colors (i.e., blue, orange, purple, and pink) convey promoter type and transcription orientation and are consistent across figures

The reporter system was constructed using our previously described GGA toolbox [14, 41]. The six biobricks and the destination vector are shown in Fig. 3a. First, donor vectors were constructed to carry *YFP*-T_LIP2_, *RedStarII*-T_LIP2_, and the *EYK1*-*EYL1* intergenic region in forward (Fw) and reverse (Rv) transcription orientations via PCR amplification. Hereinafter, the intergenic region will be referred to as pEYK450, given that it is around 450 bp long. We defined its forward orientation as being directed toward *EYK1* and its reverse orientation as being directed toward *EYL1* according to Fig. 1. Each version of pEYK450 was inserted along with the fluorescent reporter genes into the destination vector GGE114, generating plasmids GGA-*URA3*ex-*RedStarII*-pEYK450-Fw-*YFP* (JME4766) and GGA-*URA3*ex-*RedStarII*-pEYK450-Rv-*YFP* (JME4768) (Fig. 3b, Table 1). Similarly, hybrid versions of pEYK (pEYK450-5AB) were assembled, generating the plasmids GGA-*URA3*ex-*RedStarII*-pEYK450-5AB-Fw-*YFP* (JME4891) and GGA-*URA3*ex-*RedStarII*-pEYK450-5AB-Rv-*YFP* (JME4892) (Fig. 3c, Table 1, see section “Hybrid versions of pEYK450 display strong transcription levels”).

**Table 1:** Plasmids used in this study *(E. coli)*

Plasmid name	Description	Reference
*DH5α*	*Φ80dlacZΔm15, recA1, endA1, gyrA96, thi-1, hsdR17 (r*_*k*_* − , m*_*k*_ +*), supE44, relA1, deoR, Δ(lacZYA-argF)U169*	Promega
GGE114	pSB1A3-ZetaUP-*URA3*ex-RFP-ZetaDOWN, destination vector	[14]
JME3265	JMP62-*LYS5*ex	[58]
JME3935	JMP62-*URA3*ex-pEYK800-*YFP*	[21]
GGE218	GGV-pTEF-*RedStarII*-Tlip2	[22]
GGE282	GGV-pTEF-*YFP*-Tlip2	[41]
JME4760	TOPO-*RedStarII*-TLip2-reverse	This study
JME4764	TOPO-*YFP*-TLip2	This study
JME4762	TOPO-BDP-pEYK450-Fw	This study
JME4763	TOPO-BDP-pEYK450-Rv	This study
JME4766	GGA-*URA3*ex-*RedStarII*-pEYK450-Fw-*YFP*	This study
JME4768	GGA-*URA3*ex-*RedStarII*-pEYK450-Rv-*YFP*	This study
JME4859	TOPO-BDP-pEYK450-Fw, internal *Bsa*I free	This study
JME4861	TOPO-BDP-pEYK450-Rv, internal *Bsa*I free	This study
GGE0129	TOPO-pEYK300-5AB	[22]
JME4889	TOPO-BDP-pEYK450-5AB-Fw	This study
JME4890	TOPO-BDP-pEYK450-5AB-Rv	This study
JME4891	GGA-*URA3*ex-*RedStarII*-pEYK450-5AB-Fw-*YFP*	This study
JME4892	GGA-*URA3*ex-*RedStarII*-pEYK450-5AB-Rv-*YFP*	This study
GGE449	pSB1A3-ZetaUP-*URA3*ex-Tlip2(rv)-AmilCP-Txpr2(fw)-ZetaDOWN, destination vector for BDP and HBDP	This study
GGE455	TOPO-P1-pEYK450-Fw	This study
GGE456	TOPO-P1-pEYK450-5AB-Fw	This study
GGE457	TOPO-P1-pEYK450-Rv	This study
GGE458	TOPO-P1-pEYK450-5AB-Rv	This study

The suffix “-ex” in a marker indicates that it is excisable using a Cre-*lox* recombination system [57]

**Fig. 4:**
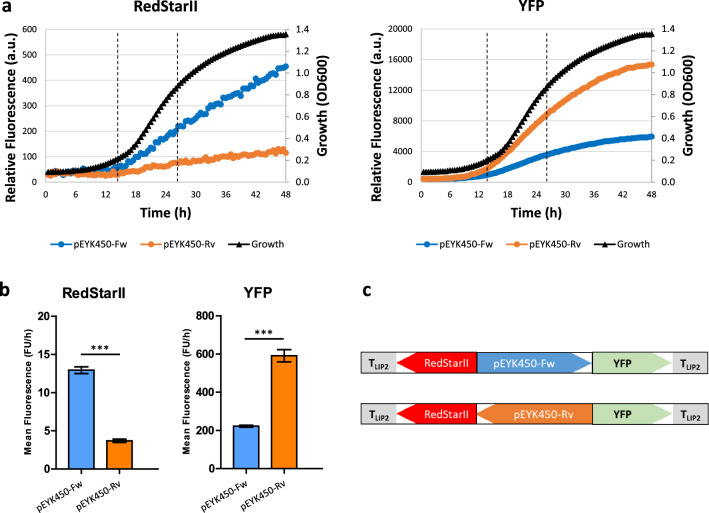
Induction levels of pEYK450 in both transcription orientations. **a** Mean RedStarII (left) and YFP (right) fluorescence produced over time by yeasts having the forward-oriented promoter (pEYK450-Fw; blue) or the reverse-oriented promoter (pEYK450-Rv; orange). Yeast were grown in YNB + 5 g/L glucose + 5 g/L erythritol. Growth curves are shown in black. Plate reader gain was set to 100 for both RedStarII and YFP. **b** Mean RedStarII (left) and YFP (right) fluorescence production rates measured during the exponential growth phase (14 h–26 h). Data represent the mean ± SEM of 8 transformants; unpaired t-test ***P < 0.0001. **c** Reporter expression cassettes with BDP contained in the strains

Resulting plasmids containing the wild-type BDP were integrated into the *Y. lipolytica* strain JMY7126. For each construct, 48 transformants were isolated. YFP and RedStarII fluorescence levels were then measured during cell growth on minimal erythritol-containing medium. Non-fluorescent transformants (*URA3* gene conversion) and highly fluorescent transformants (multiple integrations) were eliminated from the analysis. Eight representative transformants were selected for each construct and saved in our Golden Gate yeast (GGY) collection (Table 2). Fluorescence assays were performed in a microplate reader with the 16 representative transformants to assess the strength and induction levels of pEYK450 in both transcription orientations (Fig. 4).

**Table 2:** Strains used in this study *(Y. lipolytica)*

Strain name	Genotype (auxotrophy)	Reference
JMY1212	*MATA URA3-302 leu2-270-LEU2-Zeta, xpr2-322, lip2Δ, lip7Δ, lip8Δ*	[59]
JMY7126	*MATA URA3-302 leu2-270-LEU2-Zeta, xpr2-322, lip2Δ, lip7Δ, lip8Δ, lys5Δ, eyk1Δ*	[58]
GGY189 to GGY196	JMY7126 + *URA3*ex-*RedStarII*-pEYK450-Fw-*YFP* (*eyk1Δ* Ura^+^ Lys^−^)	This study
GGY197 to GGY204	JMY7126 + *URA3*ex-*RedStarII*-pEYK450-Rv-*YFP* (*eyk1Δ* Ura^+^ Lys^−^)	This study
GGY212 to GGY219	JMY7126 + *URA3*ex-*RedStarII*-pEYK450-5AB-Fw-*YFP* (*eyk1Δ* Ura^+^ Lys^−^)	This study
GGY220 to GGY227	JMY7126 + *URA3*ex-*RedStarII*-pEYK450-5AB-Rv-*YFP* (*eyk1Δ* Ura^+^ Lys^−^)	This study
GGY109	JMY7126 + *URA3*ex-pTEF-*RedStarII* (*eyk1Δ* Ura^+^ Lys^−^)	[22]
JMY7384	JMY7126 + *URA3*ex-pEYK300-*RedStarII* (*eyk1Δ* Ura^+^ Lys^−^)	[22]
JMY7392	JMY7126 + *URA3*ex-pEYK300-5AB-*RedStarII* (*eyk1Δ* Ura^+^ Lys^−^)	[22]
GGY228	JMY7126 + *URA3*ex-pTEF (*eyk1Δ* Ura^+^ Lys^−^)	This study
JMY8833	GGY212 + *LYS5*ex (*eyk1Δ* Ura^+^ Lys^+^)	This study
JMY8834	GGY220 + *LYS5*ex (*eyk1Δ* Ura^+^ Lys^+^)	This study

Yeasts containing a fluorescent reporter cassette were able to produce red and yellow fluorescence in both transcription orientations (Fig. 4a), while the control strain GGY228 showed negligible levels of fluorescence (Additional file 4: Fig. S1), confirming that pEYK450 can transcribe genes in both directions. Fluorescence varied depending on transcription orientation and showed opposite profiles. Consequently, we analyzed hourly fluorescence during the exponential growth phase and determined the promoter strength ratio between the two transcriptional directions of pEYK450. RedStarII and YFP fluorescence indicated that *EYL1*-oriented transcription was 3.5- and 2.7-fold stronger than *EYK1*-oriented transcription, respectively (Fig. 4b, c).

### pEYK450 displays dose-dependent erythritol and erythrulose induction

To determine pEYK450’s transcription characteristics, including strength and inducibility, yeast were grown in minimal media supplemented with 5 g/L of glucose as carbon source and either erythritol (0, 2.5, and 5.0 g/L) or erythrulose (0, 2.5, and 5.0 g/L) as inducer. The *Y. lipolytica* strain used in this study (JMY7126) exhibited *EYK1* gene deletion to prevent it from metabolizing erythritol and erythrulose. Given this genetic background, erythritol and erythrulose were used as inducers rather than as carbon sources. When no erythritol was added to the media, yeasts displayed neither RedStarII nor YFP fluorescence. However, when the media contained 2.5 g/L of erythritol, both RedStarII and YFP fluorescence were detected, and fluorescence levels were higher with the addition of 5.0 g/L of erythritol (Additional file 4: Fig. S1). Similar results were observed with erythrulose as the inducer (data not shown). Thus, we demonstrated that pEYK450’s erythritol and erythrulose inducibility is dose-dependent in both forward and reverse transcription orientations.

### Hybrid versions of pEYK450 exhibit strong transcription levels

Prior research showed that the hybrid promoter pEYK300-3AB, containing three copies of UAS1-eyk1 (Box A), was stronger than the native promoter pEYK300 [21]. In order to test the effect of UAS1-eyk1 on both pEYK450 transcription orientations, a new hybrid promoter was constructed by fusing five UAS1-eyk1 sequences using fusion PCR on pEYK450 and pEYK300-5AB (Additional file 4: Fig. S2). The hybrid promoter was named pEYK450-5AB because it contained five repeats of Box A and one Box B. pEYK450-5AB was inserted in both orientations along with the fluorescent reporter genes into the destination vector GGE114. Resulting plasmids JME4891 and JME4892 carried the hybrid forward-oriented promoter (pEYK450-5AB-Fw) and reverse-oriented promoter (pEYK450-5AB-Rv), respectively (Figs. 2b, 3c).

As in previous experiments, the plasmids were integrated in *Y. lipolytica,* and 48 transformants were isolated for each construct. Fluorescence levels were used to eliminate non-fluorescent transformants (*URA3* gene conversion) and highly fluorescent transformants (multiple integrations). Eight representative transformants were selected for each construct and saved in our Golden Gate yeast (GGY) collection (Table 2).

**Fig. 5:**
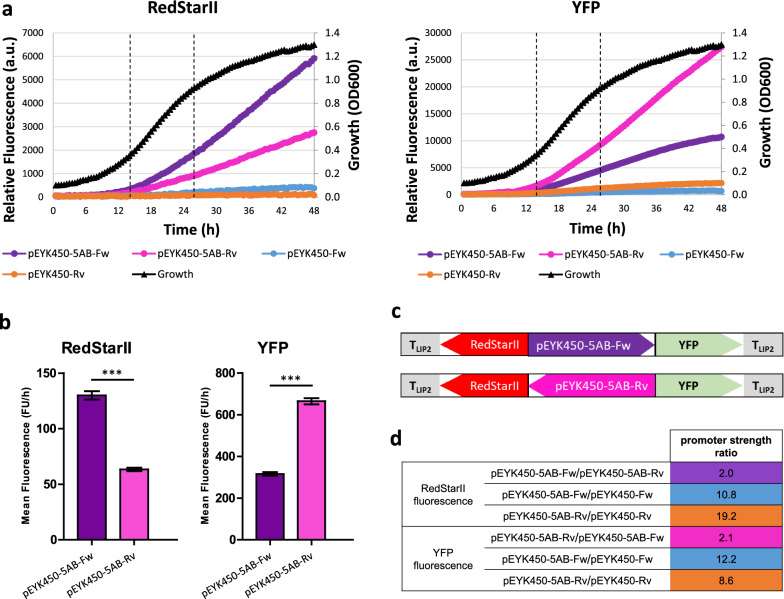
Induction levels of natural and hybrid BDP in both transcription orientations. **a** Mean RedStarII (left) and YFP (right) fluorescence produced over time by yeasts having the non-hybrid promoter in the two orientations (pEYK450-Fw = blue; pEYK450-Rv = orange) or the hybrid promoter in the two orientations (pEYK450-5AB-Fw = purple; pEYK450-5AB-Rv = pink). Yeast were grown in YNB + 5 g/L glucose + 5 g/L erythritol. Growth curves are shown in black. Plate reader gain was set to 100 for RedStarII and 77 for YFP. **b** Mean RedStarII (left) and YFP (right) fluorescence production rates measured during the exponential growth phase (14 h–26 h). Data represent the mean ± SEM of 8 transformants; unpaired t-test ***P < 0.0001. **c** Reporter expression cassettes with HBDP contained in the strains. **d** Promoter strength ratios for various transformants comparisons

Fluorescence assays were performed with 16 representative transformants containing the hybrid promoter (forward and reverse orientations) and 16 representative transformants containing the non-hybrid promoter from previous experiments. YFP and RedStarII fluorescence levels were used to quantify promoters’ strength (Fig. 5). Unexpectedly, the hybrid promoter was so strong that YFP fluorescence exceeded the measurable range, displaying a signal overflow (> 100,000 a.u.). Therefore, we lowered the gain for YFP from 100 to 77. While the scale for YFP fluorescence differs from that in the earlier experiment (Fig. 4), the strength ratios remain comparable. Fluorescence assays confirmed that the hybrid promoter pEYK450-5AB could produce RedStarII and YFP fluorescence in both transcription orientations (Fig. 5a). Moreover, pEYK450-5AB exhibited a similar strength differential between forward and reverse transcription as observed with the non-hybrid promoter. RedStarII hourly fluorescence was 63.4 FU/h with pEYK450-5AB-Rv versus 130.0 FU/h with pEYK450-5AB-Fw. Similarly, YFP hourly fluorescence was 316.1 FU/h with pEYK450-5AB-Fw versus 665.0 FU/h with pEYK450-5AB-Rv (Fig. 5b). Depending on the considered reporter gene, *EYL1*-oriented transcription was 2.0- and 2.1-fold stronger than *EYK1*-oriented transcription, which was identical to the non-hybrid promoter ratio and demonstrated that UAS1-eyk1 repeats increased transcription similarly in both directions.

Compared to the non-hybrid promoter (Fig. 5a, d), the hybrid promoter led to higher expression levels. The strength of hybrid pEYK450-5AB was up to 19.2-fold greater in the *EYK1* direction (12.2 for YFP and 19.2 for RedStarII, respectively) and up to 10.8-fold greater in the *EYL1* direction (8.6 for YFP and 10.8 for RedStarII, respectively), than the strength of non-hybrid pEYK450. Thus, it confirmed that tandem repeats of UAS1-eyk1 strongly increased promoter strength in both transcription orientations, however to a lower level toward *EYL1*.

### Hybrid pEYK450-5AB displays strong activity in both transcription orientations

**Fig. 6:**
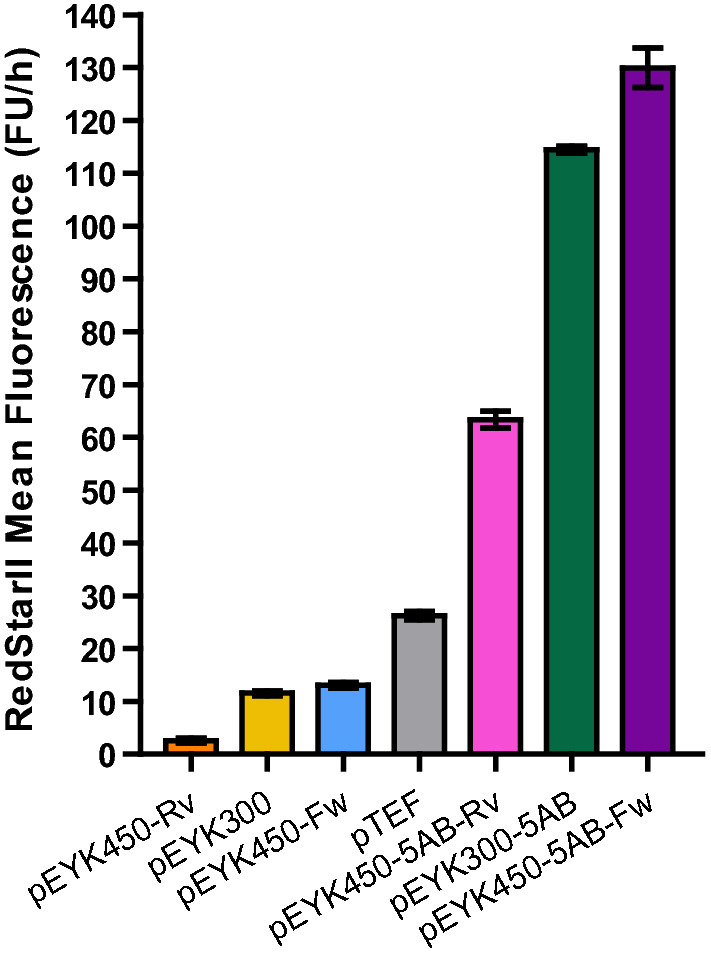
Comparison of several promoters’ strengths. Fluorescence patterns for promoters were classified in order of strength (from weakest to strongest). Mean RedStarII fluorescence production rates measured during the exponential growth phase (14 h–26 h). Yeast were grown in YNB + 5 g/L glucose + 5 g/L erythritol. Data represent the mean ± SEM of 8 transformants of strains containing pEYK450 or pEYK450-5AB, and 3 transformants of strains containing pTEF, pEYK300, and pEYK300-5AB

To further characterize the strength of pEYK450-5AB, we compared it to previously described promoters, notably the unidirectional inducible promoter pEYK300 [21] and its hybrid version pEYK300-5AB [22]. We confirmed prior research identifying that pEYK300 and pEYK450 had similar strengths [21]: pEYK300, pEYK450-Fw, and pEYK450-Rv exhibited RedStarII hourly fluorescence of 11.6, 12.0, and 3.3 FU/h, respectively (Fig. 6). Non-hybrid promoters were weaker than the constitutive promoter pTEF (26.2 FU/h), in accordance to the study of Park et al. who used the same strain *eyk1*Δ JMY7126 in similar condition [22].

In contrast, the hybrid promoters pEYK450-5AB-Rv, pEYK300-5AB, and pEYK450-5AB-Fw led to RedStarII hourly fluorescence of 63.4, 114.5 and 130.0 FU/h, respectively. Interestingly, pEYK450-5AB was 2.4-fold stronger than pTEF in the *EYK1* direction and 5.0-fold stronger in *EYL1* direction (Fig. 6).

### pEYK450-5AB strength in microbioreactors depends on media composition

We investigated whether pEYK450-5AB could be used under the high-density cultivation conditions typical of industrial settings. To this end, the strains GGY212 and GGY220 were transformed with the *LYS5*ex marker, giving rise to the prototrophic strains JMY8833 (pEYK450-5AB-Fw) and JMY8834 (pEYK450-5AB-Rv), respectively (Table 2). For 72 h, both strains were grown in microbioreactors in minimal YNB medium supplemented with 5 g/L of erythritol as the inducer and with different concentrations of glucose or glycerol.

**Fig. 7:**
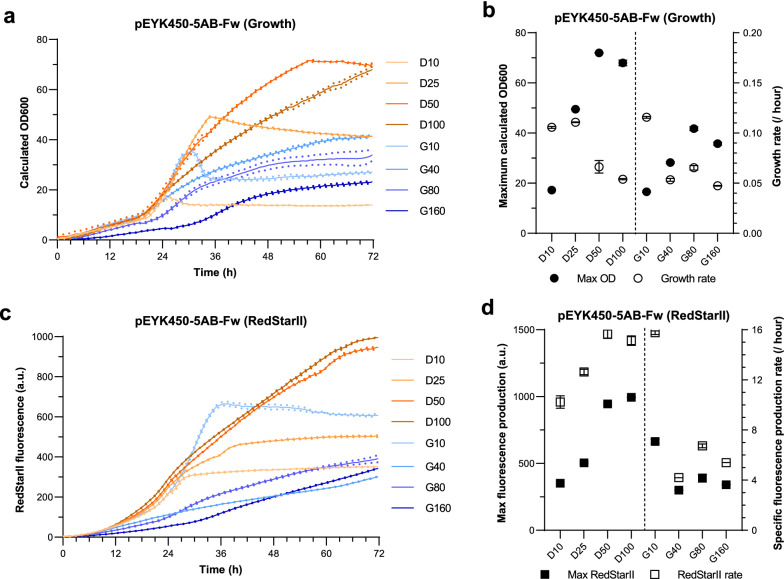
Medium optimization for growth under microbioreactor conditions.** a** Growth of JMY8833 (pEYK450-5AB-Fw) over a 72 h period in YNB medium supplemented with 5 g/L of erythritol and 10, 25, 50, or 100 g/L of glucose (treatments D10, D25, D50, and D100, respectively) or 10, 40, 80 or 160 g/L of glycerol (treatments G10, G40, G80, and G160, respectively). **b** Maximum cell density (black circles) and mean growth rate (white circles) across treatments. **c** RedStarII production over time across treatments. **d** Maximum fluorescence (black squares) and mean specific fluorescence production rate (white squares) over time across treatments. Values were standardized based on cell density and growth rate. SEM is indicated. For each condition, cultures were performed in triplicate

As expected, maximum cell density increased with glucose and glycerol concentrations, reaching a plateau at 50 g/L of glucose and 80 g/L of glycerol (Fig. 7 a, b). Although maximum cell density was the same in the medium containing 10 g/L of glucose versus glycerol, increases in glucose had a more significant impact on cell density than increases in glycerol. Maximum cell density was 1.7 times higher in glucose-based media than in glycerol-based media. Additionally, growth rates were equivalent in glucose- and glycerol-based media (≃ 0.11/h), although they were half as high when glucose concentrations exceeded 25 g/L and glycerol concentrations exceeded 10 g/L.

Maximum RedStarII fluorescence was correlated with specific fluorescence production rates across treatments. In the glucose-based media, fluorescence increased with glucose concentrations, plateauing at 50 g/L. In the glycerol-based media, fluorescence peaked at 10 g/L and then dropped by 50% at higher concentrations. Interestingly, the maximum fluorescence level reached at 10 g/L of glycerol was 2- and 1.3-fold higher than those at 10 and 25 g/L of glucose, respectively. The higher maximum fluorescence level was due to the 25–50% higher specific fluorescence production rate in the former treatment, which rivaled that seen at the highest glucose levels.

Similar conclusions could be drawn based on YFP fluorescence patterns in JMY8833, as well as RedStarII and YFP fluorescence patterns in JMY8834 (Additional file 4: Fig. S3).

### Osmotic pressure does not affect fluorescent protein production

**Fig. 8:**
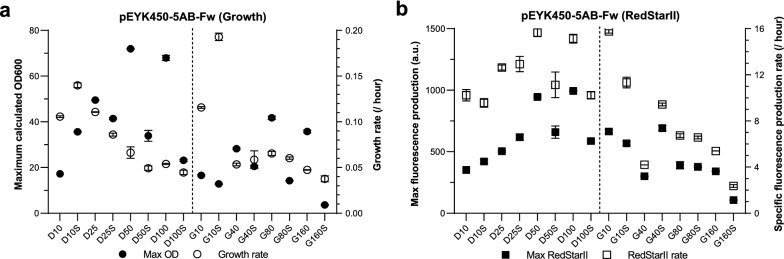
Effect of osmotic pressure on fluorescent protein production. JMY8833 (pEYK450-5AB-Fw) was cultivated for 72 h in YNB medium supplemented with 5 g/L of erythritol and 10, 25, 50, or 100 g/L of glucose (treatments D10, D25, D50, and D100, respectively) or 10, 40, 80 or 160 g/L of glycerol (treatments G10, G40, G80, and G160, respectively). High osmotic pressure was generated by adding 250 g/L of sorbitol to the media (treatments D10S, D25S, D50S, D100S, G10S, G40S, G80S, and G160S). **a** Maximum cell density (black circles) and mean growth rate (white circles) across all treatments. **b** Maximum fluorescence (black squares) and specific fluorescence production rate (white squares) for RedStarII across all treatments. Values were standardized based on cell density and growth rate. SEM is indicated. For each condition, cultures were performed in triplicate

Previous work has demonstrated that higher osmotic pressure can lead to greater erythritol production in *Y. lipolytica* [42]. We hypothesized that osmotic pressure could induce erythritol production by our strains, which could not be degraded due to the *EYK1* deletion, and would therefore result in BDP and HBDP self-activation without or reduced erythritol addition. Therefore, we investigated whether *Y. lipolytica* could self-activate pEYK450-5AB-based fluorescent protein production in response to osmotic pressure, which was generated by adding 250 g/L of sorbitol to our range of experimental media. We compared maximum cell density, mean growth rate, maximum fluorescence, and specific fluorescence production rate under high and low osmotic pressure, focusing on JMY8833 and the RedStarII reporter (Fig. 8).

In general, osmotic pressure did not impact the growth rate, except in media with low glucose or glycerol concentrations (treatments D10 and G10), where the growth rate climbed by 35 to 60%. Furthermore, the maximum cell density in D10S treatment was twice that in D10 treatment. However, when osmotic pressure was high, maximum cell density was half as great for both glucose-based and glycerol-based media; indeed, it peaked at lower concentrations of glucose and glycerol (D25S vs. D50 and G40S vs. G80). Higher glucose and glycerol concentrations were associated with lower maximum cell density under these conditions.

Osmotic pressure had no noticeable impact on RedStarII specific production rate. More interestingly, maximum fluorescence was approximately 30% greater in D10S and D25S treatments than in D10 and D25 treatments; it was 100% greater in G40S treatment versus G40 treatment. However, more broadly, maximum fluorescence was lower in the media with sorbitol than in the media without, suggesting that high osmotic pressure is detrimental to fluorescent protein production.

Again, similar conclusions could be drawn based on YFP fluorescence patterns in JMY8833 and RedStarII and YFP fluorescence patterns in JMY8834 (Additional file 4: Fig. S3).

**Fig. 9:**
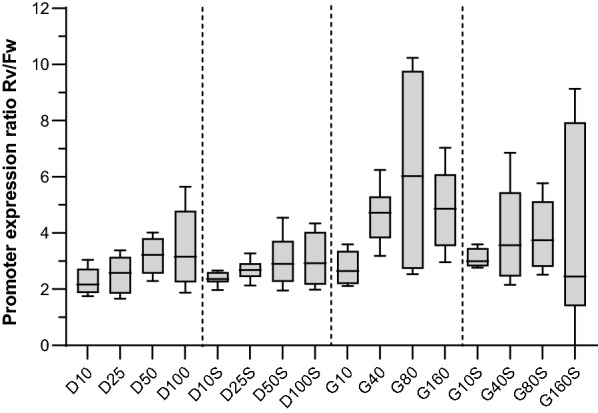
Mean strength ratio of pEYK450-5AB under several growth conditions. Strains JMY8833 (pEYK450-5AB-Fw) and JMY8834 (pEYK450-5AB-Rv) were cultivated for 72 h in YNB medium supplemented with 5 g/L of erythritol and 10, 25, 50, or 100 g/L of glucose (treatments D10, D25, D50, and D100, respectively) or 10, 40, 80, or 160 g/L of glycerol (treatments G10, G40, G80, and G160, respectively). High osmotic pressure was generated by adding 250 g/L of sorbitol to the media (treatments D10S, D25S, D50S, D100S, G10S, G40S, G80S, and G160S). The strength ratio of pEYK450-5AB in both transcription orientations was determined via four different calculations: dividing maximum RedStarII fluorescence for JMY8833 by that for JMY8834; dividing maximum YFP fluorescence for JMY8834 by that for JMY8833; dividing the specific RedStarII fluorescence production rate for JMY8833 by that for JMY8834; and dividing the specific YFP production rate for JMY8834 by that for JMY8833. The boxes show the ratio distributions across treatments

Finally, we characterized the strength ratio of pEYK450-5AB in its two transcription orientations across treatments (Fig. 9). This metric ranged between 2.3 and 6.2, displaying a mean of 3.5 and a median of 3.1, confirming previous characterization and indicating that the promoter’s strength ratio was not significantly affected by medium composition.

### Augmenting the Golden Gate toolbox for exploiting BDP and HBDP

To facilitate the use of newly constructed BDP and HBDP biobricks and allow easy assembly with other building blocks containing future genes of interest, we constructed a new destination vector specifically designed to fit our GGA strategy. The previous destination vector GGE114 was used as backbone, and the new destination vector was assembled by inserting the following fragments instead of RFP: terminator Tlip2 in reverse transcription orientation and terminator Txpr2 in forward transcription orientation separated by a gene coding for the chromoprotein AmilCP, which is used to generate blue *E. coli* colonies. The resulting destination vector was named GGE449, and it contained integration sites ZetaUP and ZetaDOWN, *URA3*ex marker for *Y. lipolytica* transformants selection, Tlip2(reverse), AmilCP, and Txpr2(forward) (Additional file 4: Fig. S4).

To ensure the compatibility of this Golden Gate destination vector and BDP biobricks for future work, the first gene (G1) should be inserted in reverse transcription orientation (3′-5′) and contain the upstream overhang ‘CTGT’ (complementing Tlip2) and the downstream overhang ‘AAGT’ (complementing the promoter). The second gene (G2) should be inserted in the forward transcription orientation (5′-3′) and contain the upstream overhang ‘TCTA’ (complementing the promoter) and the downstream overhang ‘GGAT’ (complementing Txpr2). Primers template needed to amplify and introduce *Bsa*I restriction sites with specific overhangs at the 5′ and 3′ ends of the gene of interest are indicated in Additional file 4: Table S1 (G1-GGA-with-BDP-F/R and G2-GGA-with-BDP-F/R).

In order to be also used with the classic Golden Gate kit (see [14, 41]) as unique unidirectional promoters, the four BDP biobricks were modified to introduce the required compatible overhangs. These building blocks were placed in the Promoter 1 (P1) position in unique forward or reverse transcription orientations and contained the specific overhangs ‘ACGG’ at the 5′ end and ‘AATG’ at the 3′ end. Thus, they are ready to use with the classical destination vector GGE114.

## Discussion

Promoters are key elements in gene expression systems, either for producing recombinant proteins, modulating biosynthetic pathways, or co-expressing proteins. One important promoter characteristic is inducibility, which is commonly performed by the media carbon source [43]. For example, galactose-inducible promoters have been frequently deployed in *Saccharomyces cerevisiae* [44]. Additionally, tight D-amino acid-inducible promoters have been described in *Rhodosporidium/Rhodoturola* species [45]. To date, erythritol-inducible promoters have been developed for *Y. lipolytica* [21].

The development of hybrid promoters combining a core promoter, a TATA box, and multiple UAS copies has allowed fine-tuning of heterologous gene expression in yeast [46]. This approach has been applied to create strong regulated promoters in *Y. lipolytica* [19, 20, 22, 35]. For example, a hybrid phase-dependent promoter was built with UAS1B-xpr2 [19], and hybrid erythritol-inducible promoters were generated with UAS-eyk1 [22].

Great interest in BDPs has emerged as researchers seek to improve gene co-expression for metabolic engineering and synthetic biology applications, as revealed by recent BDPs utilization in *E. coli* [47], *S. cerevisiae* [48], plants [49], and mammals [50]. However, studies on yeast BDPs remain scarce, and few BDPs have been developed, especially for non-conventional yeasts. A BDPs set was generated for the yeast *Komagataella phaffii* (syn. *Pichia pastoris*) [51], and, more recently, copper-inducible BDPs were identified and engineered for *Y. lipolytica* [35].

In this study, we identified the first erythritol-inducible BDP in *Y. lipolytica* and used it to build a strong HBDP by inserting multiple copies of UAS-eyk1. By revisiting the genetic structure of the erythritol utilization cluster, we demonstrated that the *EYK1*-*EYL1* intergenic region (pEYK450) fulfilled the requirements to be a BDP. Through further analysis, we identified a previously missed TATA box (Box TATA 2) upstream of *EYK1*.

To experimentally validate the bidirectional nature of pEYK450, we designed a GGA strategy for constructing a dual fluorescent reporter cassette. Additionally, we constructed a new destination vector containing the chromoprotein AmilCP instead of the chromoprotein RFP [14, 41] to augment the GGA toolbox and simplify the selection of *E. coli* transformants. This new destination vector was specifically designed to fit our BDP and HBDP biobricks. It will facilitate the use of BDPs for co-expressing and co-regulating two genes of interest.

By performing fluorescence assays, we confirmed the bidirectional nature of pEYK450 and demonstrated that transcription is erythritol inducible in both directions. We showed that pEYK450’s strength was around threefold greater when oriented toward *EYL1* than toward *EYK1*. Interestingly, the strength ratio remained identical after inserting multiple copies of UAS-eyk1. To enhance clarity in future studies, we recommend referring to pEYK450 as pEYL1 and pEYK450-5AB as pEYL1-5AB when used in reverse transcription orientation (toward *EYL1*).

Furthermore, we demonstrated that pEYK450-5AB’s strength ratio remained close to 3 in every tested medium, demonstrating its stability under various growth conditions. How the ratio was calculated can explain the apparent pronounced variation (Fig. 9); we provided four different calculation methods, each of which has a margin of error. The latter was higher under weak growth conditions because the exponential phase was hard to determine.

We have identified that yeast growth was negatively affected by high concentrations of glucose, glycerol, or sorbitol, possibly because cells were experiencing osmotic stress or difficulties dividing in highly viscous environments. The growth decrease was correlated with a fluorescent protein production deficit, which was not offset by *Yarrowia lipolytica*’s potential for self-activation under high osmotic pressure conditions. Nonetheless, we demonstrated that pEYK450-5AB remained functional under the typical high-density cultivation conditions of industrial settings. We found that the best fluorescent protein production medium, in terms of cell growth, maximum production, and production rate, was YNB with 50 g/L of glucose. Importantly, the highest production rate was reached in YNB containing 10 g/L of glycerol, with a 25% lower fluorescent protein production despite a four times lower cell density caused by medium carbon source exhaustion. These findings suggest that the best fluorescent protein production strategy — in terms of yield and productivity — would be to perform fed-batch cultivation with regular additions of glycerol (≤ 10 g/L). Further investigation under bioreactor conditions would be necessary to validate this hypothesis.

While multiple UAS copies can increase a promoter’s strength [19, 20, 22, 35, 52, 53], it may increase transcriptional leakage under non-induced conditions [35]. We demonstrated that pEYK450-5AB did not exhibit such leakage.

In the classical GGA strategy, where three transcription units can have the same promoter, loss of expression units and pathway inactivation could occur because of intra-cassette recombination. Recombination frequency could even increase when using hybrid promoters because of longer homologous sequences coming from multiple UAS copies. When the diversity of available promoters is weak, using BDPs is a way of drastically reducing recombination frequency.

Unidirectional promoter strength could be varied via promoter shuffling to optimize pathway operation, as shown with β-carotene production in *Y. lipolytica*. The best performance was seen when pTEF was combined with three transcription units [54]. *GND1* and *ZWF1*, genes allowing the NADPH cofactor to be regenerated [55], or *ERG20* and *IDI*, genes showed to improve β-carotene production in *Y. lipolytica* [56], have been co-expressed successfully using BDP to boost β-carotene production even further (Vidal L., Robles R., unpublished).

In summary, bidirectional promoters developed here offer a convenient way to co-express two genes with several strengths in *Y. lipolytica*. In future studies, BDPs and HBDPs could limit the accumulation of toxic metabolites by generating balanced protein stoichiometry.

## Conclusions

Bidirectional promoters characterized in this study have great potential for synthetic biology applications with *Y. lipolytica*, such as highly coordinated co-expression of multi-gene enzymatic pathways. They can ensure that key proteins, metabolites, and cofactors are supplied in appropriate stoichiometric ratios.

## Methods

### Plasmid and yeast strain construction

#### Plasmid and biobrick construction

The template and constructed plasmids are described in Table 1, and the PCR primer pairs are described in Additional file 4: Table S1. The *Escherichia coli DH5α* strain was used for plasmid propagation. Restriction enzymes and T4 DNA ligase were obtained from New England Biolabs (NEB, MA, USA). PCR amplifications were performed using an Applied Biosystems 2720 Thermal Cycler, with Q5® High-Fidelity DNA Polymerase (NEB) for amplification purposes and with GoTaq® DNA Polymerase (Promega, WI, USA) for construction verification. Restriction enzymes, ligase, and DNA polymerases were used in accordance with the manufacturer’s recommendations. Plasmids were isolated using a NucleoSpin Plasmid EasyPure Kit (Machery-Nagel, Duren, Germany), and PCR fragments were purified using a NucleoSpin Gel and PCR Clean-up Kit (Machery-Nagel). DNA sequencing was carried out by Eurofins Genomics (Ebersberg, Germany). Benchling software was used for gene sequence analysis and primer design. All the biobricks were constructed according to the Golden Gate assembly strategy, exploiting *Bsa*I overhangs (Fig. 3) and GGA toolbox, as described elsewhere [41].

#### Construction of pEYK450-Fw and pEYK450-Rv promoters

The source of the pEYK450-Fw and pEYK450-Rv promoters was plasmid JME3935, which came from our laboratory’s *E. coli* collection and which contains the intergenic region between *EYL1* and *EYK1*. Plasmids containing the two promoters were produced via PCR amplification using the primer pairs forwardpEYK450-F/forwardpEYK450-R and reversepEYK450-F/reversepEYK450-R, which were designed to introduce the *Bsa*I restriction sites with specific overhangs at the 5′ and 3′ ends of the amplified fragment. This process created a 460 bp fragment carrying Box TATA 1, Box A, Box B, and Box TATA 2, within *Bsa*I sites, in both the forward (pEYK450-Fw) and reverse (pEYK450-Rv) transcription orientations (see Figs. 2a ,  3). These fragments were cloned into pCR Blunt II-TOPO donor vector (Thermo Fisher Scientific, Villebon sur Yvette, France), yielding the plasmids JME4762 (TOPO-pEYK450-Fw) and JME4763 (TOPO-pEYK450-Rv), which were verified by *Bsa*I digestion and sequencing.

#### Construction of the hybrid pEYK450 promoters: pEYK450-5AB-Fw and pEYK450-5AB-Rv

Fusion PCR was performed to introduce the four additional copies of UAS1-eyk1 (Box A). First, an internal *Bsa*I site was deleted in JME4762 and JME4763 via mutagen PCR with the primer pair pEYK450-internal-F/pEYK450-internal-R, a procedure that yielded *Bsa*I-free pEYK450-Fw and pEYK450-Rv plasmids (*E. coli* strains JME4859 and JME4861, respectively). Second, the hybrid pEYK450 promoters —pEYK450-5AB-Fw and pEYK450-5AB-Rv— were constructed using fusion PCR (Additional file 4: Fig. S2). The resulting *E. coli* strains JME4889 and JME4890 carried the pEYK450-5AB promoter in the forward (pEYK450-5AB-Fw) and reverse (pEYK450-5AB-Rv) transcription orientations (see Fig. 2b, 3).

#### Construction of RedStarII and YFP biobricks

*RedStarII* and *YFP* were used as reporter genes to analyze BDP expression. We constructed *RedStarII* and *YFP* fragments with the Lip2 terminator by purifying plasmids from our Golden Gate *E. coli* (GGE) collection (Table 1) and then carrying out amplification using the primers designed to have the upstream and the downstream overhangs compatible with the GGA assembly strategy described in Fig. 3. The plasmid carrying *RedStarII*-TLip2 was constructed to have the reverse transcription orientation (JME4760), and the plasmid carrying *YFP*-TLip2 was constructed to have the forward transcription orientation (JME4764). Construction success was verified using *Bsa*I digestion and sequencing.

#### Plasmid construction by Golden Gate assembly

All the primers used to amplify the promoters were designed to have the upstream overhang ‘AAGT’ and the downstream overhang ‘TCTA’ after *Bsa*I digestion (Additional file 4: Table S1 and Fig. 3); these overhangs are instrumental in our newly designed GGA strategy. Other GGA building blocks (RedStarII, YFP, with Lip2 terminator) were designed to be compatible with the BDP and the overhangs ‘ACGG’ and ‘GAGT’ of the destination vector. The destination vector GGE114, pSB1A3-ZetaUP-*URA3*ex-RFP-ZetaDOWN contained the following components: ZetaUP, *URA3*ex marker, RFP (red fluorescent protein, which can be used to generate red *E. coli* colonies) and ZetaDOWN (Fig. 3a). The Golden Gate reaction conditions have been described previously [14, 41]. The reaction mixture contained predetermined equimolar amounts of each Golden Gate biobrick and destination vector (50 pmol); it also included 1.5 µL of T4 DNA ligase buffer (NEB), 400 U of T4 DNA ligase (NEB), 10 U of *Bsa*I (NEB), and up to 15 µL of ddH2O. The following thermal profile was applied: 37 °C for 5 min, 16 °C for 5 min for 60 cycles, 50 °C for 5 min, 80 °C for 5 min, and 15 °C ∞. The reaction mixture was then used for *E. coli* DH5α transformation. White colonies were screened for the presence of the final assembly. PCR and *Not*I digestion were conducted on purified plasmids for verification purposes.

#### Novel destination vector and Golden Gate biobricks construction for BDPs utilization

A new destination vector for BDP and HBDP utilization was constructed with a specific design according to our GGA strategy. The previous destination vector GGE114 –containing ZetaUP, *URA3*ex marker, RFP, and ZetaDOWN– was used as the backbone, and its RFP fragment was replaced with the following fragments: terminator Tlip2 in reverse transcription orientation, terminator Txpr2 in forward transcription orientation, and, in between both, the gene coding for the chromoprotein AmilCP. The fragment containing a codon-optimized version of AmilCP (BBa_K2669002, iGEM Registry of Standard Biological Parts) has been synthesized (GeneCust, France). The obtained destination vector was named GGE449 (Additional file 4: Fig. S4). On the other hand, so that they could also be used with the classic GGA kit as unique unidirectional promoters, the four BDP biobricks were modified to introduce the required compatible overhangs ‘ACGG’ at the 5′ end and ‘AATG’ at the 3’ end. These biobricks, now in Promoter 1 (P1) position, were referred to as GGE455 through GGE458 (Table 1).

#### Construction of *Y*. *lipolytica* strains

The *eyk1*Δ strain JMY7126 was derived from the *EYK1* WT strain JMY1212 via successive gene deletion and marker rescue [58]. The plasmids constructed for the BDP and HBDP experiments were digested by *Not*I, which allowed the expression cassette to be released from the vector prior to JMY7126 transformation. Transformation of yeast cells employed 400 ng of DNA and the lithium acetate method [60]. Transformants were then selected on YNB+lysine medium based on their genotypes. Integration of the expression cassette was verified via colony PCR with specific primers (Additional file 4: Table S1). Fluorescence assays were carried out for 48 transformants obtained from each construct; 8 representative clones were then chosen for further analysis. The strains containing the expression cassettes with pEYK450-Fw, pEYK450-Rv, hybrid pEYK450-5AB-Fw, or hybrid pEYK450-5AB-Rv were named GGY189 to GGY196, GGY197 to GGY204, GGY212 to GGY219, and GGY220 to GGY227, respectively. To construct prototrophic strains, the LYS5 fragment from plasmid JME3265 was transformed, and these strains were named JMY8833 and JMY8834 (Table 2).

### Growth media and culture conditions

The *E. coli DH5α* strain was used to host and amplify the recombinant plasmid DNA. All the strains used in this study are listed in Table 1. The *E. coli* strains were grown at 37 °C in Lysogeny Browth (LB) medium supplemented with either kanamycin sulfate (50 µg/mL) or ampicillin (100 µg/mL). The *Y. lipolytica eyk1*Δ strain, JMY7126, was used in this study. Growth of *Y. lipolytica* was performed at 28 °C in rich medium (YPD) or minimal glucose medium (YNB), which were prepared as described below. The YPD medium contained 10 g/L of yeast extract (Difco, Paris, France), 10 g/L of Peptone (Difco, Paris, France), and 10 g/L of glucose (Sigma Aldrich, Saint-Quentin Fallavier, France). The YNB medium contained 1.7 g/L of yeast nitrogen base without amino acids and ammonium sulfate (YNBww; BD Difco, Paris, France), 5.0 g/L of NH_4_Cl, and 50 mM phosphate buffer (pH 6.8). It was supplemented with either 10 g/L (transformants selection in plate) or 5 g/L of glucose (culture assays in BioTek microplate reader). To meet the auxotrophic requirement, lysine (0.8 g/L) was added to the culture medium as necessary. Solid media were created by adding 1.5% agar.

### Growth in microplate and fluorescence analysis

#### Growth and fluorescence analysis in the microplate reader

*Y. lipolytica* pre-cultures were grown overnight in YNB+lysine with 5 g/L of glucose. Microplates (96-well) containing 200 µL (final volume) of the appropriate medium were inoculated with cells at an OD_600nm_ of 0.1. YNB+lysine medium supplemented with glucose (5 g/L) as the carbon source and either erythritol (0, 2.5, or 5.0 g/L) or erythrulose (0, 2.5, or 5.0 g/L) as the inducer, was used for the growth and fluorescence analysis. The strains were kept at 28 °C with constant shaking in a Synergy Mx microplate reader (BioTek, Colmar, France) in accordance with the manufacturer’s instructions. OD_600nm_, YFP fluorescence, and RedStarII fluorescence were measured every 30 min for 48 h; Gen5 software was used for detection purposes. Red fluorescence was analyzed at the following wavelength settings: excitation at 558 nm, emission at 586 nm, gain at 100. Yellow fluorescence was analyzed at the following wavelength settings: excitation at 505 nm, emission at 530 nm, gain between 77 and 100, which was adjusted to obtain optimal setting for reading value within the dynamic range. RedStarII and YFP fluorescence were expressed in arbitrary units. Fluorescence levels were expressed in mean fluorescence units per hour (FU/h) and the standard error of the mean (SEM). The fluorescence production rate was calculated during the exponential phase for each treatment group. Statistical analyses were performed using GraphPad Prism software. Statistical significance was determined via two-tailed unpaired t-tests. P < 0.05 was considered statistically significant. The control strains used in this study are listed in Table 2 and came from our GGY and JMY collection. All control strains had the same parental strain, JMY7126, carried different promoters, and either included *RedStarII* as reporter or had no reporter. Cultures were performed in triplicate, for which eight biological replicates were used.

#### Growth and fluorescence analysis in the microbioreactor

*Y. lipolytica* pre-cultures were grown overnight in YPD. FlowerPlates (m2p-labs, MTP-48-BOH 1) containing 800 µL (final volume) of the appropriate medium were inoculated with cells at an OD_600nm_ of 0.1. YNB medium supplemented with glucose (10, 25, 50, or 100 g/L, respectively designated as treatments D10, D25, D50, and D100) or glycerol (10, 40, 80, or 160 g/L, respectively designated as treatments G10, G40, G80, and G160) as the carbon source, erythritol (5 g/L) as the inducer, was used for the growth and fluorescence analysis. Then, these same media sets were complemented with sorbitol (250 g/L) to test the impacts of osmotic pressure on growth and fluorescence (treatments D10S, D25S, D50S, D100S, G10S, G40S, G80S, and G160S). The strains were kept at 28 °C, 1200 rpm, and 85% humidity in a BioLector I microbioreactor (m2p-labs). The scattered light at 620 nm, pH, pO2, and YFP/RedStarII fluorescence intensities were measured every 30 min for 72 h. Red fluorescence was analyzed at the following wavelength settings: excitation at 550 nm, emission at 580 nm, gain at 100. Yellow fluorescence was analyzed at the following wavelength settings: excitation at 508 nm, emission at 532 nm, gain at 40. Biomass was calculated using OD_600mn_ values and a calibration curve (Additional file 4: Fig. S5). RedStarII and YFP fluorescence levels were expressed in arbitrary units. Mean growth rate was calculated between the beginning and end of the exponential growth phase. To calculate the mean specific fluorescence production rate, we divided the fluorescence signal measured at each time point by cell density and the mean growth rate. Once again, the rate was calculated between the beginning and end of the exponential growth phase. The standard error of the mean (SEM) was also determined. The strains used in this study are listed in Table 2. Cultures were performed in triplicate.

### Bioinformatics analyses

The chromosomal sequences for the erythritol genes cluster were retrieved for three *Y. lipolytica* strains: CLIB89/W29 (CP028453; NCBI), A101 (YALIA101S02; GRYC), and CLIB122/E150 (YALI0F; GRYC). For each strain, we retained the genetic region containing the erythritol genes cluster starting 1 kb upstream of the first gene (YALI0F01540 in E150) and finishing 1 kb downstream of the last gene (YALI0F01672 in E150). The resulting sequences were named E150_F(2238146–266510), W29_F(226377–254838), and A101_S02(2920929–2892546) (Additional files 1, 2, 3). First, the E150 sequence was annotated in SnapGene using the information provided by GRYC. Then, E150 was used as a reference to annotate in-CDS nucleotide and amino-acid variation in W29 and A101. A search for the undiscovered ORF was performed with Open Reading Frame Finder [61].

Multiple sequence alignments of the intergenic region between *EYL1* and *EYK1* within the *Yarrowia* clade (*Y. lipolytica*, *Y. phangngensis*, *Y. yakushimensis*, *Y. alimentaria* and *Y. galli*) were reanalyzed from previously published data [21] with Clustal Omega software [62].

## Supplementary Information

**Additional file 1: **Yarrowia-E150-F(238146-262831).gb: annotated sequence of the erythritol locus in E150 strain in chromosome F coordinate 238146-262831.

**Additional file 2: **Yarrowia-W29-F(226377-254838).gb: annotated sequence of the erythritol locus in W29 strain in chromosome F coordinate 226377-254838.

**Additional file 3: **Yarrowia-A101-S02(2920929-2892546).gb: annotated sequence of the erythritol locus in A101 strain in chromosome F coordinate 2920929-2892546.

**Additional file 4: Table S1. **List of primers used in this study. **Figure S1**. Patterns of erythritol-induced fluorescence depending on the inducer level. **Figure S2.** Construction of the hybrid promoters pEYK450-5AB-Fw and pEYK450-5AB-Rv. **Figure S3.** Patterns of fluorescence for strains grown under microbioreactor conditions. **Figure S4.** Schematic representation of the Golden Gate assembly strategy proposed for exploiting BDP and HBDP. **Figure S5.** Calibration curve for estimating *Y. lipolytica* biomass from scattered light and OD600nm values.

## Data Availability

Data sharing is not relevant as no datasets were generated or analyzed during the current study.

## Abbreviations

AmilCP: Blue chromoprotein (codon-optimized for *E. coli*)
BDP: Bidirectional promoter
bp: DNA base pair
D: Glucose
*EYK1*: Erythrulose kinase encoding gene
*EYL1*: Erythrulose-4-phosphate isomerase encoding gene
FU: Fluorescence unit
F: Forward primer
Fw: Forward transcription
G: Glycerol
GGA: Golden Gate Assembly
GGE: *E. coli* Laboratory Golden Gate strain collection
GGY: *Y. lipolytica* Laboratory Golden Gate strain collection
h: Hour
HBDP: Hybrid bidirectional promoter
JME: *E. coli* Laboratory strain collection
JMY: *Y. lipolytica* Laboratory strain collection
Lys/LYS5: Saccharopine dehydrogenase [NAD( +), L-lysine-forming]
PCR: Polymerase chain reaction
R: Reverse primer
Rv: Reverse transcription
RFP: Red Fluorescent Protein (codon-optimized for *E. coli*)
RedStarII: Red fluorescent protein derived from RFP (codon-optimized for *Y. lipolytica*)
S: Sorbitol
UAS: Upstream activating sequence
Ura/URA3: Orotidine-5'-phosphate decarboxylase
YFP: Yellow Fluorescent Protein (codon-optimized for *Y. lipolytica*)
YNB: Yeast Nitrogen Base

## References

[1] Nicaud JM, Madzak C, Van Den Broek P, Gysler C, Duboc P, Niederberger P (2002). Protein expression and secretion in the yeast *Yarrowia lipolytica*. FEMS Yeast Res.

[2] Madzak C (2015). *Yarrowia lipolytica*: recent achievements in heterologous protein expression and pathway engineering. Appl Microbiol Biotechnol.

[3] Dulermo R, Brunel F, Dulermo T, Ledesma-Amaro R, Vion J, Trassaert M (2017). Using a vector pool containing variable-strength promoters to optimize protein production in *Yarrowia lipolytica*. Microbial Cell Fact BioMed Central.

[4] Holz M, Otto C, Kretzschmar A, Yovkova V, Aurich A, Pötter M (2011). Overexpression of alpha-ketoglutarate dehydrogenase in *Yarrowia lipolytica* and its effect on production of organic acids. Appl Microbiol Biotechnol.

[5] Rywińska A, Juszczyk P, Wojtatowicz M, Rymowicz W (2011). Chemostat study of citric acid production from glycerol by *Yarrowia lipolytica*. J Biotechnol.

[6] Rymowicz W, Rywińska A, Marcinkiewicz M (2009). High-yield production of erythritol from raw glycerol in fed-batch cultures of *Yarrowia lipolytica*. Biotech Lett.

[7] Carly F, Vandermies M, Telek S, Steels S, Thomas S, Nicaud JM (2017). Enhancing erythritol productivity in *Yarrowia lipolytica* using metabolic engineering. Metab Eng.

[8] Pagot Y, Le Clainche A, Nicaud J-M, Wache Y, Belin J-M (1998). Peroxisomal β-oxidation activities and γ-decalactone production by the yeast *Yarrowia lipolytica*. Appl Microbiol Biotechnol.

[9] Gomes N, Teixeira JA, Belo I (2010). The use of methyl ricinoleate in lactone production by *Yarrowia lipolytica*: aspects of bioprocess operation that influence the overall performance. Biocatal Biotransform Taylor Francis.

[10] Celińska E, Olkowicz M, Grajek W (2015). L-Phenylalanine catabolism and 2-phenylethano synthesis in *Yarrowia lipolytica*-mapping molecular identities through whole-proteome quantitative mass spectrometry analysis. FEMS Yeast Res..

[11] Park Y-K, González-Fernández C, Robles-Iglesias R, Vidal L, Fontanille P, Kennes C (2021). Bioproducts generation from carboxylate platforms by the non-conventional yeast *Yarrowia lipolytica*. FEMS Yeast Res.

[12] Madzak C (2018). Engineering *Yarrowia lipolytica* for use in biotechnological applications: a review of major achievements and recent innovations. Mol Biotechnol.

[13] Park Y-K, Ledesma-Amaro R (2022). What makes *Yarrowia lipolytica* well suited for industry?. Trends Biotechnol.

[14] Celińska E, Ledesma-Amaro R, Larroude M, Rossignol T, Pauthenier C, Nicaud JM (2017). Golden gate assembly system dedicated to complex pathway manipulation in *Yarrowia lipolytica*. Microb Biotechnol.

[15] Schwartz CM, Hussain MS, Blenner M, Wheeldon I (2016). Synthetic RNA polymerase III promoters facilitate high-efficiency CRISPR–Cas9-mediated genome editing in *Yarrowia lipolytica*. ACS Synth Biol.

[16] Schwartz C, Shabbir-Hussain M, Frogue K, Blenner M, Wheeldon I (2017). Standardized markerless gene Integration for pathway engineering in *Yarrowia lipolytica*. ACS Synth Biol.

[17] Wong L, Engel J, Jin E, Holdridge B, Xu P (2017). YaliBricks, a versatile genetic toolkit for streamlined and rapid pathway engineering in *Yarrowia lipolytica*. Metabolic Eng Commun.

[18] Beneyton T, Thomas S, Griffiths AD, Nicaud JM, Drevelle A, Rossignol T (2017). Droplet-based microfluidic high-throughput screening of heterologous enzymes secreted by the yeast *Yarrowia lipolytica*. Microbial Cell Fact BioMed Central.

[19] Madzak C, Tréton B, Blanchin-Roland S (2000). Strong hybrid promoters and integrative expression/secretion vectors for quasi-constitutive expression of heterologous proteins in the yeast *Yarrowia*
*lipolytica*. J Mol Microbiol Biotechnol.

[20] Shabbir Hussain M, Gambill L, Smith S, Blenner MA (2016). Engineering promoter architecture in oleaginous yeast *Yarrowia lipolytica*. ACS Synth Biol.

[21] Trassaert M, Vandermies M, Carly F, Denies O, Thomas S, Fickers P (2017). New inducible promoter for gene expression and synthetic biology in *Yarrowia lipolytica*. Microb Cell Fact.

[22] Park Y-K, Korpys P, Kubiak M, Celinska E, Soudier P, Trébulle P (2019). Engineering the architecture of erythritol-inducible promoters for regulated and enhanced gene expression in *Yarrowia lipolytica*. FEMS Yeast Res.

[23] Larroude M, Rossignol T, Nicaud JM, Ledesma-Amaro R (2018). Synthetic biology tools for engineering *Yarrowia lipolytica*. Biotechnol Adv.

[24] Ganesan V, Spagnuolo M, Agrawal A, Smith S, Gao D, Blenner M (2019). Advances and opportunities in gene editing and gene regulation technology for *Yarrowia lipolytica*. Microbial Cell Fact.

[25] Ma J, Gu Y, Marsafari M, Xu P (2020). Synthetic biology, systems biology, and metabolic engineering of *Yarrowia lipolytica* toward a sustainable biorefinery platform. J Ind Microbiol Biotechnol.

[26] Carly F, Gamboa-Melendez H, Vandermies M, Damblon C, Nicaud JM, Fickers P (2017). Identification and characterization of *EYK1*, a key gene for erythritol catabolism in *Yarrowia lipolytica*. Appl Microbiol Biotechnol.

[27] Carly F, Steels S, Telek S, Vandermies M, Nicaud JM, Fickers P (2018). Identification and characterization of *EYD1*, encoding an erythritol dehydrogenase in *Yarrowia lipolytica* and its application to bioconvert erythritol into erythrulose. Bioresource Technol Elsevier.

[28] Rzechonek DA, Neuvéglise C, Devillers H, Rymowicz W, Mirończuk AM (2017). *EUF1*-A newly identified gene involved in erythritol utilization in *Yarrowia lipolytica*. Sci Rep.

[29] Mirończuk AM, Biegalska A, Zugaj K, Rzechonek DA, Dobrowolski A (2018). A role of a newly identified isomerase from *Yarrowia lipolytica* in erythritol catabolism. Front Microbiol.

[30] Niang PM, Arguelles-Arias A, Steels S, Denies O, Nicaud JM, Fickers P (2020). In *Yarrowia lipolytica* erythritol catabolism ends with erythrose phosphate. Cell Biol Intern.

[31] Bagchi DN, Iyer VR (2016). The determinants of directionality in transcriptional initiation. Trends Genet.

[32] Sammarco MC, Grabczyk E (2005). A series of bidirectional tetracycline-inducible promoters provides coordinated protein expression. Anal Biochem.

[33] Rendsvig JKH, Workman CT, Hoof JB (2019). Bidirectional histone-gene promoters in *Aspergillus*: characterization and application for multi-gene expression. Fungal Biol Biotechnol.

[34] Yang S, Sleight SC, Sauro HM (2013). Rationally designed bidirectional promoter improves the evolutionary stability of synthetic genetic circuits. Nucleic Acids Res.

[35] Xiong X, Chen S (2020). Expanding toolbox for genes expression of *Yarrowia lipolytica* to include novel inducible, repressible, and hybrid promoters. ACS Synth Biol.

[36] Dujon B, Sherman D, Fischer G, Durrens P, Casaregola S, Lafontaine I (2004). Genome evolution in yeasts. Nature.

[37] Magnan C, Yu J, Chang I, Jahn E, Kanomata Y, Wu J (2016). Sequence assembly of *Yarrowia lipolytica* strain W29/CLIB89 shows transposable element diversity. PLoS ONE.

[38] Tomaszewska-Hetman L, Rymowicz W, Rywińska A (2020). Waste conversion into a sweetener—development of an innovative strategy for erythritol production by *Yarrowia lipolytica*. Sustainability.

[39] Wolf T, Shelest V, Nath N, Shelest E (2016). CASSIS and SMIPS: promoter-based prediction of secondary metabolite gene clusters in eukaryotic genomes. Bioinformatics.

[40] Bagchi DN, Iyer VR (2016). The determinants of directionality in transcriptional initiation. Trends Genet.

[41] Larroude M, Park Y-K, Soudier P, Kubiak M, Nicaud JM, Rossignol T (2019). A modular golden gate toolkit for *Yarrowia lipolytica* synthetic biology. Microb Biotechnol.

[42] Yang L-B, Zhan X-B, Zheng Z-Y, Wu J-R, Gao M-J, Lin C-C (2014). A novel osmotic pressure control fed-batch fermentation strategy for improvement of erythritol production by *Yarrowia lipolytica* from glycerol. Biores Technol.

[43] Weinhandl K, Winkler M, Glieder A, Camattari A (2014). Carbon source dependent promoters in yeasts. Microb Cell Fact.

[44] Peng B, Wood RJ, Nielsen LK, Vickers CE (2018). An expanded heterologous *GAL* promoter collection for Diauxie-Inducible expression in *Saccharomyces cerevisiae*. ACS Synth Biol.

[45] Liu Y, Koh CMJ, Ngoh ST, Ji L (2015). Engineering an efficient and tight d-amino acid-inducible gene expression system in *Rhodosporidium/Rhodotorula* species. Microb Cell Fact.

[46] Blazeck J, Garg R, Reed B, Alper HS (2012). Controlling promoter strength and regulation in *Saccharomyces cerevisiae* using synthetic hybrid promoters. Biotechnol Bioeng.

[47] Christoffersen CA, Brickman TJ, Hook-Barnard I, McIntosh MA (2001). Regulatory architecture of the iron-regulated *fepD-ybdA* bidirectional promoter region in *Escherichia coli*. J Bacteriol.

[48] Öztürk S, Ergün BG, Çalık P (2017). Double promoter expression systems for recombinant protein production by industrial microorganisms. Appl Microbiol Biotechnol.

[49] Lv X, Song X, Rao G, Pan X, Guan L, Jiang X (2009). Construction vascular-specific expression bi-directional promoters in plants. J Biotechnol.

[50] Léjard V, Rebours E, Meersseman C, Rocha D (2014). Construction and validation of a novel dual reporter vector for studying mammalian bidirectional promoters. Plasmid.

[51] Vogl T, Kickenweiz T, Pitzer J, Sturmberger L, Weninger A, Biggs BW (2018). Engineered bidirectional promoters enable rapid multi-gene co-expression optimization. Nat Commun.

[52] Blazeck J, Liu L, Redden H, Alper H (2011). Tuning Gene Expression in *Yarrowia lipolytica* by a Hybrid Promoter Approach. Appl Environ Microbiol.

[53] Blazeck J, Reed B, Garg R, Gerstner R, Pan A, Agarwala V (2013). Generalizing a hybrid synthetic promoter approach in *Yarrowia lipolytica*. Appl Microbiol Biotechnol.

[54] Larroude M, Celinska E, Back A, Thomas S, Nicaud JM, Ledesma-Amaro R (2018). A synthetic biology approach to transform *Yarrowia lipolytica* into a competitive biotechnological producer of β-carotene. Biotechnol Bioeng.

[55] Cheng H, Wang S, Bilal M, Ge X, Zhang C, Fickers P (2018). Identification, characterization of two NADPH-dependent erythrose reductases in the yeast *Yarrowia lipolytica* and improvement of erythritol productivity using metabolic engineering. Microb Cell Fact.

[56] Arnesen JA, Kildegaard KR, Cernuda Pastor M, Jayachandran S, Kristensen M, Borodina I (2020). *Yarrowia lipolytica* strains engineered for the production of terpenoids. Front Bioeng Biotechnol.

[57] Fickers P, Le Dall MT, Gaillardin C, Thonart P, Nicaud JM (2003). New disruption cassettes for rapid gene disruption and marker rescue in the yeast *Yarrowia lipolytica*. J Microbiol Methods.

[58] Park Y-K, Vandermies M, Soudier P, Telek S, Thomas S, Nicaud JM (2019). Efficient expression vectors and host strain for the production of recombinant proteins by *Yarrowia lipolytica* in process conditions. Microbial Cell Fact.

[59] Bordes F, Fudalej F, Dossat V, Nicaud JM, Marty A (2007). A new recombinant protein expression system for high-throughput screening in the yeast *Yarrowia lipolytica*. J Microbiol Methods.

[60] Barth G, Gaillardin C, Wolf K (1996). Nonconventional Yeasts in Biotechnology. Yarrowia lipolytica.

[61] NCBI Software Tool to Search for Open Reading Frames (ORF) in the DNA Sequence Open Reading Frame Finder (RRID:SCR_016643). https://www.ncbi.nlm.nih.gov/orffinder.

[62] Clustal Omega (RRID:SCR_001591) Multiple Sequence Alignment. EMBL-EBI. https://www.ebi.ac.uk/Tools/msa/clustalo/.

