# Supplementary material for: Bidirectional hybrid erythritol-inducible promoter for synthetic biology in Yarrowia lipolytica
Source: Microb Cell Fact. 2023 Jan 12;22:7. doi: 10.1186/s12934-023-02020-6 (PMC9835291; doi:10.1186/s12934-023-02020-6)
Supplement: Supplementary file 4 — Additional file 4: Table S1. List of primers used in this study. Figure S1. Patterns of erythritol-induced fluorescence depending on the inducer level. Figure S2. Construction of the hybrid promoters pEYK450-5AB-Fw and pEYK450-5AB-Rv. Figure S3. Patterns of fluorescence for strains grown under microbioreactor conditions. Figure S4. Schematic representation of the Golden Gate assembly strategy proposed for exploiting BDP and HBDP. Figure S5. Calibration curve for estimating Y. lipolytica biomass from scattered light and OD600nm values. [file 12934_2023_2020_MOESM4_ESM.docx]

Additional file 4

**Bidirectional hybrid erythritol-inducible promoter for synthetic biology in *Yarrowia lipolytica***

Lea Vidal^1^*, Esteban Lebrun^1,2^*, Young-Kyoung Park^1^, Guillaume Mottet^2^ and Jean-Marc Nicaud^1ǂ^

**Author affiliations**

^1^Université Paris-Saclay, INRAE, AgroParisTech, Micalis Institute, 78350 Jouy-en-Josas, France

^2^Large Molecules Research, Sanofi, 94400 Vitry-Sur-Seine, France

*These authors contributed equally to this work.

**ǂCorresponding author**

Université Paris-Saclay, INRAE, AgroParisTech, Micalis Institute, UMR1319, Team BIMLip: Integrative Biology of Microbial Lipid Metabolism, domaine de Vilvert, 78350 Jouy-en-Josas, France

Tel: +33 1 74 07 18 20

Email: jean-marc.nicaud@inrae.fr

**Keywords:** Bidirectional promoter, Inducible, Erythritol, Hybrid promoter, co-expression, *Yarrowia lipolytica*, Synthetic biology

**Additional Table S1**

***Additional Table S1 List of primers used in this study***

The bases corresponding to the *Bsa*I recognition site are underlined; the overhang bases generated after digestion are in **bold**; nnn corresponds to the overlap of at least 20 nucleotides with the sequence to be amplified. F: forward primer; R: reverse primer.

| **Primer** | **Sequence (5’-3’)** | **Utilization** |
| --- | --- | --- |
| forwardpEYK450-F | GGTCTCT**AAGT**TTTGTGCAAGTGTGTGTGTGTGTGTGTG | Amplification for biobrick construction |
| forwardpEYK450-R | GGTCTCT**TAGA**AGTAGATGTGTAAGTGTGTAGAAGTGTCG | Amplification for biobrick construction |
| reversepEYK450-F | GGTCTCT**AAGT**AGTAGATGTGTAAGTGTGTAGAAGTGTCGTG | Amplification for biobrick construction |
| reversepEYK450-R | GGTCTCT**TAGA**TTTGTGCAAGTGTGTGTGTGTGTGTGTG | Amplification for biobrick construction |
| reverseRedStarII-F | GGTCTCT**ACGG**TGTCTTAGAGGAACGCATATACAGTAATCATAG | Amplification for biobrick construction |
| reverseRedStarII-R | GGTCTCT**ACTT**ATGAGTGCTTCTTCTGAAGATGTCATCACTG | Amplification for biobrick construction |
| YFP-F | GGTCTCT**TCTA**ATGGTGAGCAAGGGCGAGGAGCTGTTC | Amplification for biobrick construction |
| YFP-R | GGTCTCT**ACTC**TGTCTTAGAGGAACGCATATACAGTAATC | Amplification for biobrick construction |
| G1-GGA-with-BDP-F | GGTCTCT**CTGT**nnn | Amplification for biobrick construction |
| G1-GGA-with-BDP-R | GGTCTCT**ACTT**nnn | Amplification for biobrick construction |
| G2-GGA-with-BDP-F | GGTCTCT**TCTA**nnn | Amplification for biobrick construction |
| G2-GGA-with-BDP-R | GGTCTCT**ATCC**nnn | Amplification for biobrick construction |
| P1-forwardpEYK450-F | GGTCTCT**ACGG**TTTGTGCAAGTGTGTGTGTGTGTGTG | Amplification for biobrick construction |
| P1-forwardpEYK450-R | GGTCTCT**CATT**AGTAGATGTGTAAGTGTGTAGAAGTGTCG | Amplification for biobrick construction |
| P1-reversepEYK450-F | GGTCTCT**ACGG**AGTAGATGTGTAAGTGTGTAGAAGTGTCG | Amplification for biobrick construction |
| P1-reversepEYK450-R | GGTCTCT**CATT**TTTGTGCAAGTGTGTGTGTGTGTGTG | Amplification for biobrick construction |
| pEYK450-internal-F | GTGTGAGTGTGTAGTTGTGTGATGAGATCTTGGTGCCACCCCAAGGTATATATATATAAC | Removal of internal *Bsa*I site |
| pEYK450-internal-R | GTTATATATATATACCTTGGGGTGGCACCAAGATCTCATCACACAACTACACACTCACAC | Removal of internal *Bsa*I site |
| pEYK-boxA-F | GCATCTACTTTTCTCTATACTGTACGTTTCAATCTGGG | Fusion PCR |
| pEYK-boxA-R | CCCAGATTGAAACGTACAGTATAGAGAAAAGTAGATGC | Fusion PCR |
| ZetaUP-internal-F | TATCTTCTGACGCATTGACCAC | Verification of Golden Gate assembly |
| URA3-internal-F | CATCCAGAGAAGCACACAGG | Verification of Golden Gate assembly |
| RedStarII-internal-F | AAGACGGTGGCGTTGTTACT | Verification of Golden Gate assembly |
| RedStarII-internal-R | GACTTGCTTCTTGGCCTTGT | Verification of Golden Gate assembly |
| YFP-internal-F | ACGTAAACGGCCACAAGTTC | Verification of Golden Gate assembly |
| YFP-internal-R | AAGTCGTGCTGCTTCATGTG | Verification of Golden Gate assembly |
| ZetaDOWN-internal-R | GGTAACGCCGATTCTCTCTG | Verification of Golden Gate assembly |

**Additional Figure S1**


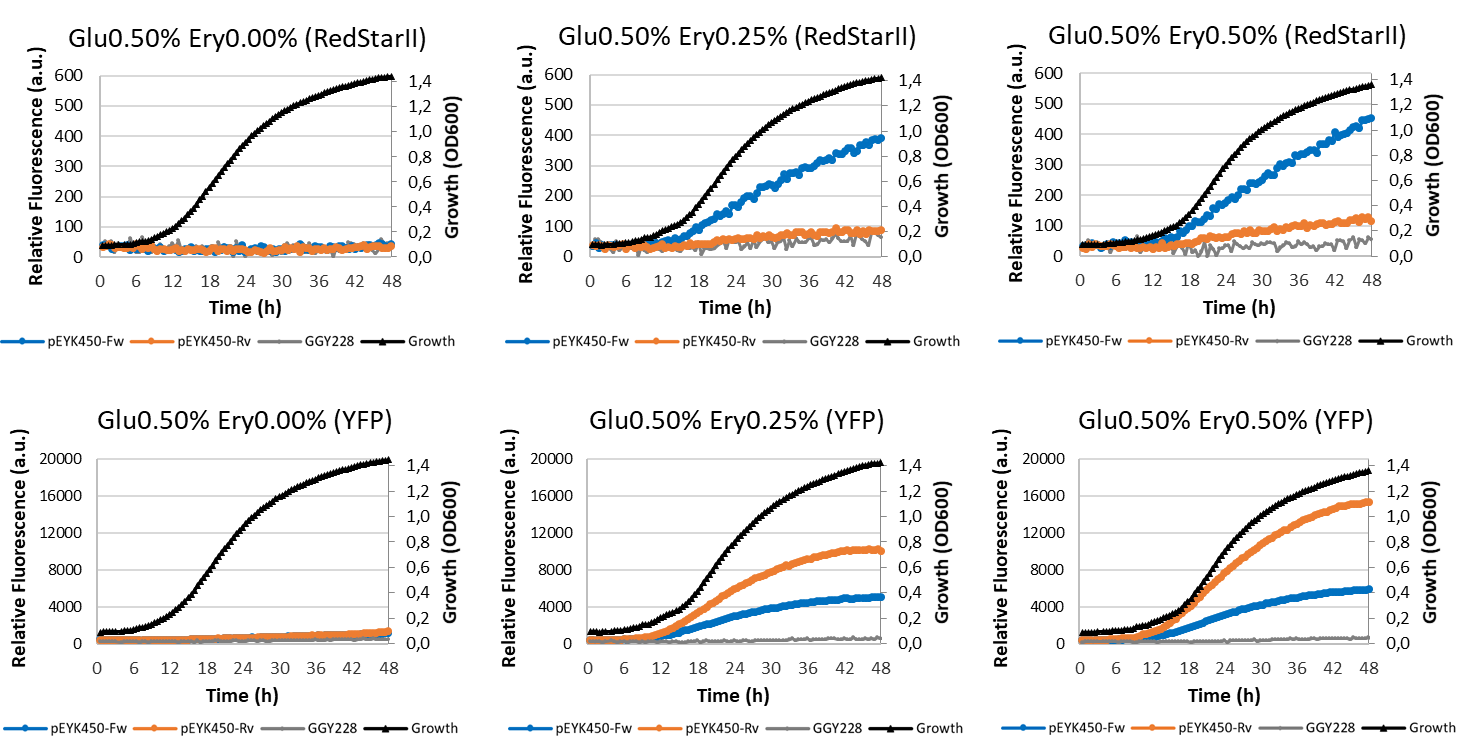


***Additional Fig. S1 Patterns of erythritol-induced fluorescence.*** *Mean RedStarII (above) and YFP (below) fluorescence over time depending on the inducer level. The blue (pEYK450-Fw) and orange (pEYK450-Rv) curves represent the mean fluorescence measured for 8 transformants. The gray curves represent the mean fluorescence of the control strain (GGY228), which does not contain a fluorescent reporter gene. The gain was 100 for both RedStarII and YFP. Yeast were grown in minimum media containing 5 g/L of glucose (Glu0.50%) as the carbon source and 0.0, 2.5 and 5.0 g/L of erythritol (Ery0.00%, Ery0.25% and Ery0.50%, respectively) as the inducer. The growth curves are shown in black.*

**Additional Figure S2**


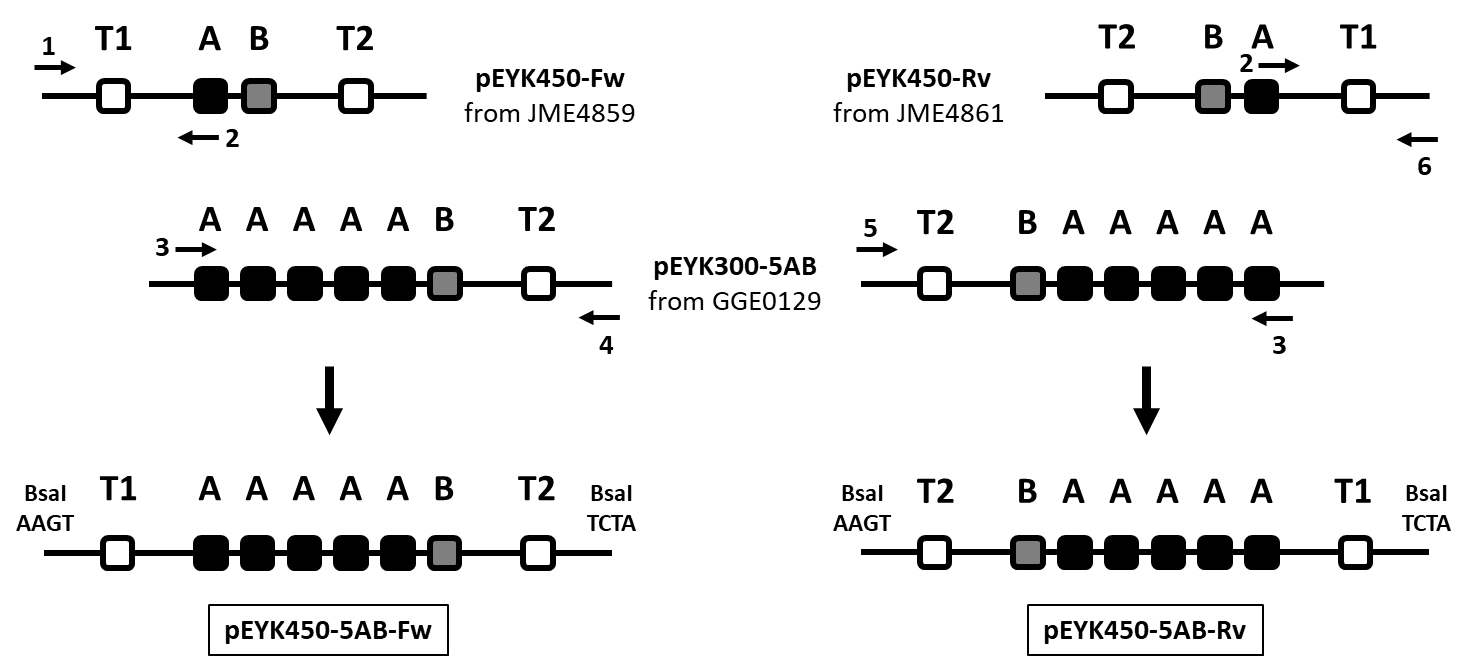


***Additional Fig. S2 Construction of the hybrid promoters pEYK450-5AB-Fw and pEYK450-5AB-Rv.*** *The upstream T1 A block and the downstream 5A-B-T2 block were amplified and fused to generate the T1-5AB-T2 fragment, which carried the BsaI sites with their AAGT and TCTA compatible overhangs. In this way, we created two versions of the hybrid promoter pEYK450-5AB: one with forward-oriented transcription (Fw) and one with reverse-oriented transcription (Rv). The following primers were used—1: forwardpEYK450-F, 2: pEYK-boxA-R, 3: pEYK-boxA-F, 4: forwardpEYK450-R, 5: reversepEYK450-F, and 6: reversepEYK450-R (Additional Table S1). The template plasmids employed during PCR are indicated in Table 1.*

**Additional Figure S3**


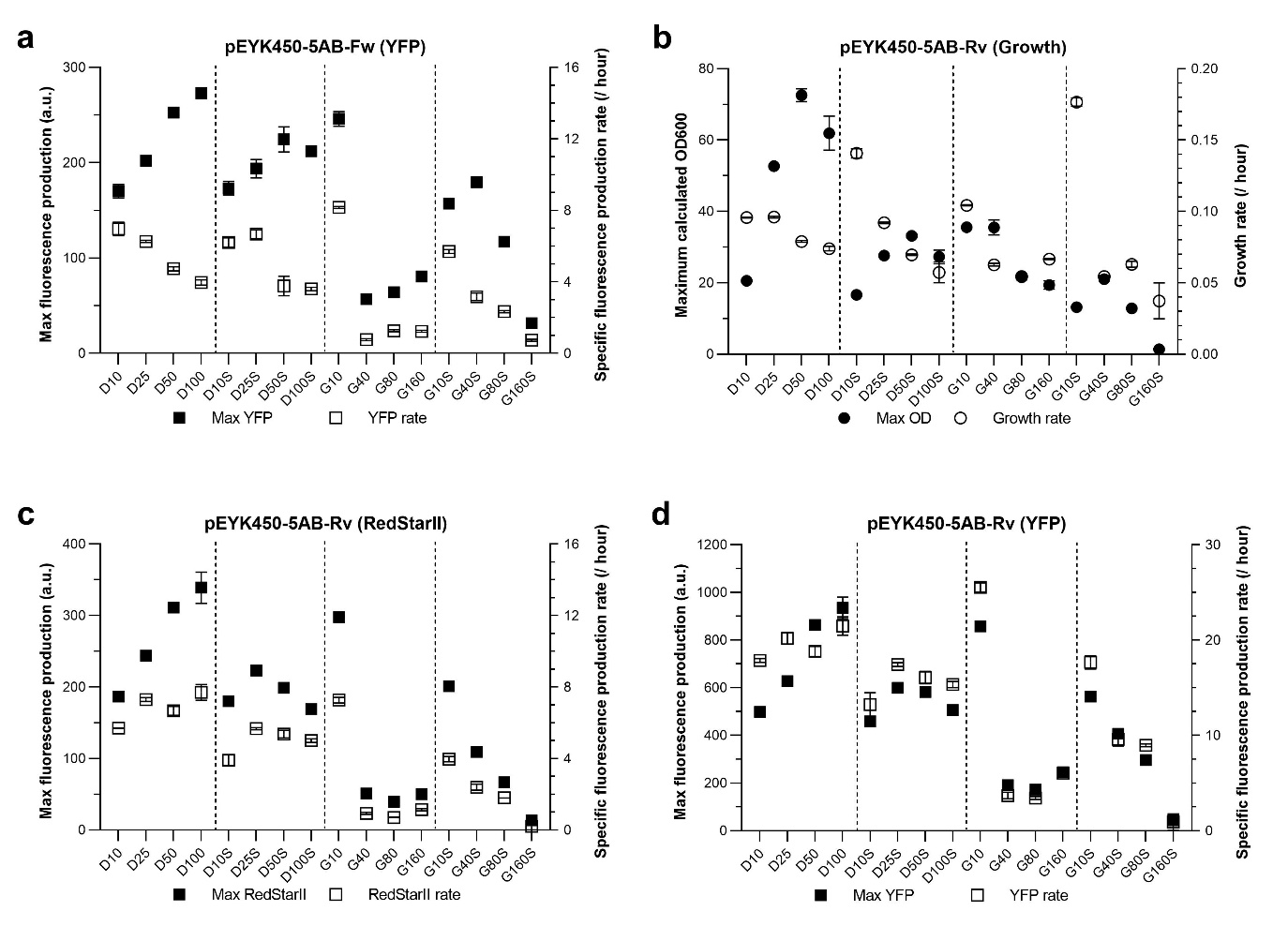


***Additional Fig. S3 Patterns of fluorescence for strains grown under microbioreactor conditions.*** *Strains JMY8833 (pEYK450-5AB-Fw) and JMY8834 (pEYK450-5AB-Rv) were cultivated for 72 h in YNB medium supplemented with 5 g/L of erythritol and with 10, 25, 50, or 100 g/L of glucose (treatments D10, D25, D50, and D100, respectively) or 10, 40, 80 or 160 g/L of glycerol (treatments G10, G40, G80, and G160, respectively). High osmotic pressure was generated by adding 250 g/L of sorbitol to the media (treatments D10S, D25S, D50S, D100S, G10S, G40S, G80S, and G160S).* ***a*** *Maximum fluorescence (black squares) and specific fluorescence production rate (white squares) for YFP displayed by JMY8833 (pEYK450-5AB-Fw) across all treatments.* ***b*** *Maximum cell density (black circles) and mean growth rate (white circles) for JMY8834 (pEYK450-5AB-Rv) across all treatments.* ***c*** *Maximum fluorescence (black squares) and specific fluorescence production rate (white squares) for RedStarII displayed by JMY8834 (pEYK450-5AB-Rv) across all treatments.* ***d*** *Maximum fluorescence (black squares) and specific fluorescence production rate (white squares) for YFP displayed by JMY8834 (pEYK450-5AB-Rv) across all treatments. Values were standardized based on cell density and growth rate. The standard error of the mean (SEM) is indicated. For each condition, cultures were performed in triplicate.*

**Additional Figure S4**


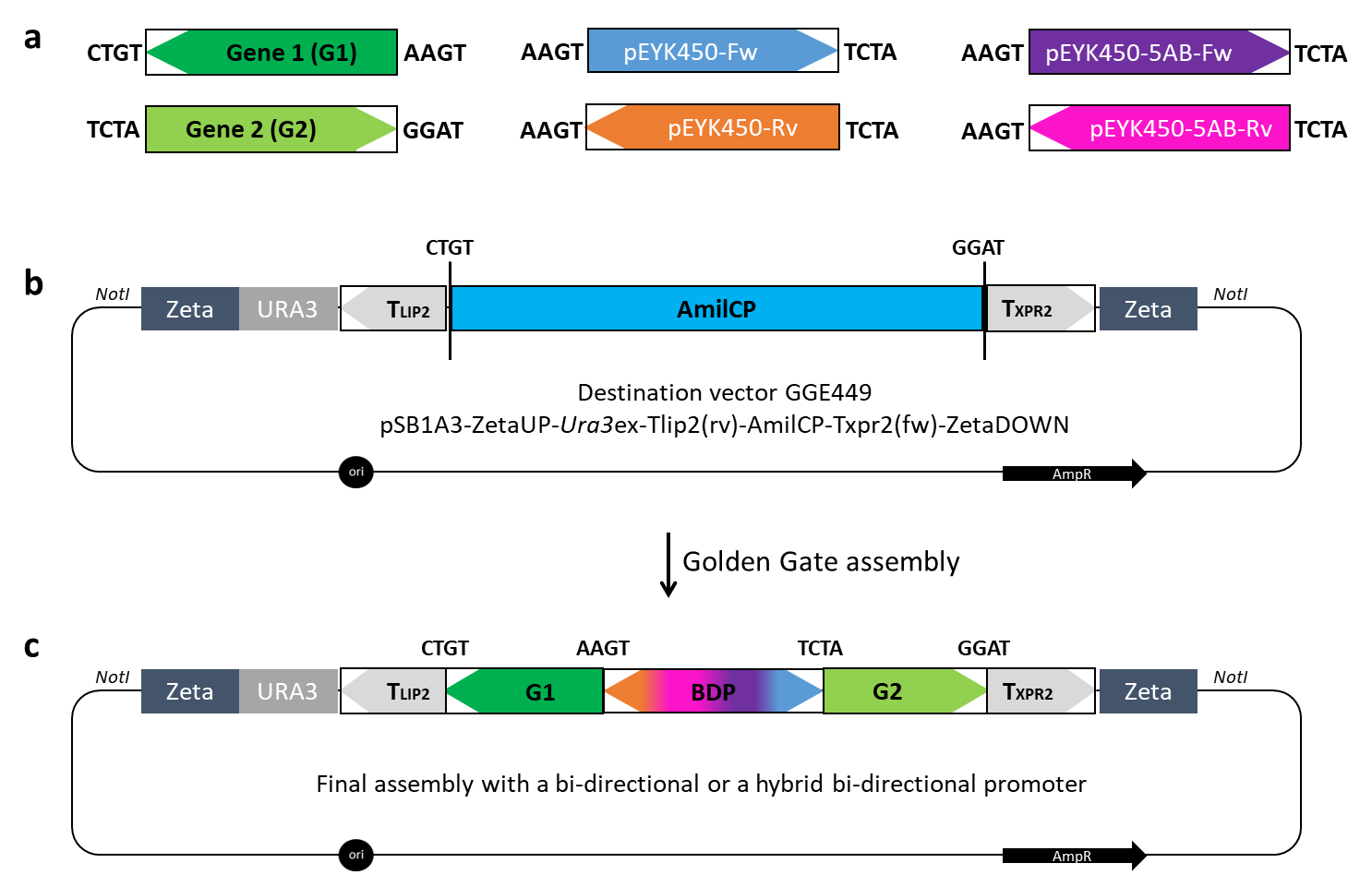


***Additional Fig. S4 Schematic representation of the Golden Gate assembly strategy proposed for exploiting BDP and HBDP. a*** *The four available BDP-bearing biobricks and the two biobricks to be constructed that will carry a gene of interest. The letters to either side of the biobricks represent the four-nucleotide overhangs generated by the BsaI restriction sites. To be compatible with this GGA strategy, the first gene (G1) should be oriented in reverse transcription orientation (3’-5’) and designed with the upstream overhang CTGT and the downstream overhang AAGT; the second gene (G2) should be oriented in forward transcription orientation (5’-3’) and designed with the upstream overhang TCTA and the downstream overhang GGAT.* ***b*** *The destination vector GGE449 designed to be used with a BDP or an HBDP. It contains the specific integration sites ZetaUP and ZetaDOWN, the URA3 marker, the T_LIP2_ and T_XPR2_ terminators, and the gene coding for the blue chromophore AmilCP.* ***c*** *Schematic representation of the final vector obtained after GGA. The biobrick containing AmilCP, surrounded by the GGA digestion/ligation sites, has been replaced by the constructed cassette, which contains two genes of interest that are co-expressed by one of the BDP or HBDP. The expression cassette can be released by NotI digestion to promote yeast transformation.*

**Additional Figure S5**


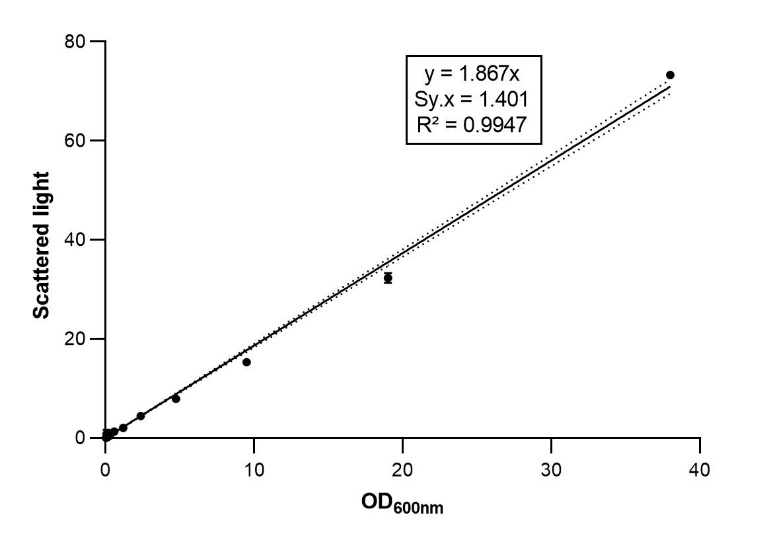


***Additional Fig. S5 Calibration curve for estimating* Y. lipolytica *biomass from scattered light and OD_600nm_ values.*** *Using serial dilutions of an overnight culture (200 µL per plate well), we measured OD_600nm_ using an Ultrospec 10 Cell Density Meter and scattered light using a BioLector I microbioreactor. The SEM is indicated.*
